# Evaluating the Genetics of Common Variable Immunodeficiency: Monogenetic Model and Beyond

**DOI:** 10.3389/fimmu.2018.00636

**Published:** 2018-05-14

**Authors:** Guillem de Valles-Ibáñez, Ana Esteve-Solé, Mònica Piquer, E. Azucena González-Navarro, Jessica Hernandez-Rodriguez, Hafid Laayouni, Eva González-Roca, Ana María Plaza-Martin, Ángela Deyà-Martínez, Andrea Martín-Nalda, Mónica Martínez-Gallo, Marina García-Prat, Lucía del Pino-Molina, Ivón Cuscó, Marta Codina-Solà, Laura Batlle-Masó, Manuel Solís-Moruno, Tomàs Marquès-Bonet, Elena Bosch, Eduardo López-Granados, Juan Ignacio Aróstegui, Pere Soler-Palacín, Roger Colobran, Jordi Yagüe, Laia Alsina, Manel Juan, Ferran Casals

**Affiliations:** ^1^Institut de Biologia Evolutiva (UPF-CSIC), Departament de Ciències Experimentals i de la Salut, Universitat Pompeu Fabra, Parc de Recerca Biomèdica de Barcelona, Barcelona, Spain; ^2^Allergy and Clinical Immunology Department, Hospital Sant Joan de Déu, Institut de Recerca Pediàtrica Hospital Sant Joan de Déu, Barcelona, Spain; ^3^Functional Unit of Clinical Immunology Hospital Sant Joan de Déu-Hospital Clinic, Barcelona, Spain; ^4^Servei d’Immunologia, Centre de Diagnòstic Biomèdic, Hospital Clinic-IDIBAPS, Barcelona, Spain; ^5^Bioinformatics Studies, ESCI-UPF, Barcelona, Spain; ^6^Pediatric Infectious Diseases and Immunodeficiencies Unit, Hospital Universitari Vall d’Hebron (HUVH), Vall d’Hebron Institut de Recerca (VHIR), Universitat Autònoma de Barcelona, Barcelona, Spain; ^7^Jeffrey Modell Diagnostic and Research Center for Primary Immunodeficiencies, Barcelona, Spain; ^8^Immunology Division, Department of Clinical and Molecular Genetics, Hospital Universitari Vall d’Hebron (HUVH), Vall d’Hebron Research Institute (VHIR), Barcelona, Spain; ^9^Department of Cell Biology, Physiology and Immunology, Universitat Autònoma de Barcelona, Barcelona, Spain; ^10^Clinical Immunology Department, University Hospital La Paz and Physiopathology of Lymphocytes in Immunodeficiencies Group, IdiPAZ Institute for Health Research, Madrid, Spain; ^11^Department of Experimental and Health Sciences, Universitat Pompeu Fabra, Barcelona, Spain; ^12^Centro de Investigación Biomédica en Red de Enfermedades Raras (CIBER-ER), Madrid, Spain; ^13^Servei de Genòmica, Departament de Ciències Experimentals i de la Salut, Universitat Pompeu Fabra, Parc de Recerca Biomèdica de Barcelona, Barcelona, Spain; ^14^Catalan Institution of Research and Advanced Studies (ICREA), Barcelona, Spain; ^15^CNAG-CRG, Centre for Genomic Regulation, Barcelona Institute of Science and Technology (BIST), Barcelona, Spain

**Keywords:** common variable immunodeficiency, primary immunodeficiency, exome sequencing, loss-of-function, rare disease genetics

## Abstract

Common variable immunodeficiency (CVID) is the most frequent symptomatic primary immunodeficiency characterized by recurrent infections, hypogammaglobulinemia and poor response to vaccines. Its diagnosis is made based on clinical and immunological criteria, after exclusion of other diseases that can cause similar phenotypes. Currently, less than 20% of cases of CVID have a known underlying genetic cause. We have analyzed whole-exome sequencing and copy number variants data of 36 children and adolescents diagnosed with CVID and healthy relatives to estimate the proportion of monogenic cases. We have replicated an association of CVID to p.C104R in TNFRSF13B and reported the second case of homozygous patient to date. Our results also identify five causative genetic variants in *LRBA, CTLA4, NFKB1*, and *PIK3R1*, as well as other very likely causative variants in *PRKCD, MAPK8*, or *DOCK8* among others. We experimentally validate the effect of the *LRBA* stop-gain mutation which abolishes protein production and downregulates the expression of CTLA4, and of the frameshift indel in *CTLA4* producing expression downregulation of the protein. Our results indicate a monogenic origin of at least 15–24% of the CVID cases included in the study. The proportion of monogenic patients seems to be lower in CVID than in other PID that have also been analyzed by whole exome or targeted gene panels sequencing. Regardless of the exact proportion of CVID monogenic cases, other genetic models have to be considered for CVID. We propose that because of its prevalence and other features as intermediate penetrancies and phenotypic variation within families, CVID could fit with other more complex genetic scenarios. In particular, in this work, we explore the possibility of CVID being originated by an oligogenic model with the presence of heterozygous mutations in interacting proteins or by the accumulation of detrimental variants in particular immunological pathways, as well as perform association tests to detect association with rare genetic functional variation in the CVID cohort compared to healthy controls.

## Introduction

Common variable immunodeficiency (CVID) is the most prevalent symptomatic primary humoral immunodeficiency with a prevalence from 1:10,000 to 1:50,000 in North America and Europe (1). The diagnosis criteria consist in low serum concentrations of IgG, IgA and/or IgM, recurrent bacterial infections and poor antibody response to vaccines, in addition to the exclusion of other known causes of hypogammaglobulinemia (1–4). Patients’ phenotypes are highly heterogeneous due to different time onsets and to a high variety of related complications, such as autoimmune manifestations, lymphoproliferation, enteropathy, and lymphoid malignancies, suggesting that CVID could be a common outcome of diverse immune system failures.

The clinical heterogeneity of CVID has hindered both the diagnostic and the identification of the underlying genetic defect of the disease, allowing a molecular characterization of the origin in less than 20% of the patients, and usually in familiar forms of the disease which constitute only a small fraction of the CVID cases (1, 5–7). Despite that, mutations in the genes *CR2, LRBA, NFKB1, NFKB2, IL21, TNFRSF13B, TNFRSF13C, CD81, IKZF1, PRKCD, MS4A1*, and *CD19* are listed in the OMIM database^1^ as causative of disease, inducing reclassification of CVID in these new diagnostics, and establishing new therapeutic approaches based on the affected pathways that have markedly improved affected patients’ prognoses (8). Specific variants in these genes as well as in others not listed in the OMIM database (*NOD2, MSH5, TNFRSF13B, HLA*) have been reported to confer susceptibility to the disease or to originate similar phenotypes to CVID (*CTLA4, PLCG2, PIK3CD, PIK3R1*), blurring even more the boundaries that define this disorder. Furthermore, some of the mutations have incomplete penetrance (9, 10) and many sporadic cases remain unexplained after deep genetic analyzes, suggesting that an important fraction of CVID cases might not follow a monogenic Mendelian pattern of inheritance (11).

In recent studies using whole-genome and exome sequencing to study CVID, 15–30% of CVID patients have been proposed to have a monogenic origin (12–14), with genetic variants both at candidate or new genes for CVID, although not all of these mutations have been functionally validated. In this work, we aim to estimate the proportion of monogenic cases in CVID and to explore other possible genetic models for CVID. For that, we have analyzed high coverage whole-exome sequencing and copy number variants data for 36 CVID pediatric patients. We hypothesize that focusing on pediatric cases will allow us to estimate the maximum proportion of monogenic CVID cases, based on the higher incidence of infectious disease in childhood and theoretical and molecular evidence of higher impact of inborn single gene defects in childhood than in adults, which tend to present more complex genetics of predisposition to infection (15, 16). Because of the heterogeneity of CVID etiology and manifestations, we first examined the role of known genetic variants and candidate genes for CVID, and then expanded the analysis to interacting proteins and genes in the same pathway, and finally to the rest of the genome. We propose single candidate genes for the CVID patients according to different models of inheritance and by considering both genetic variants properties such as the allele frequency, bioinformatic predictions of the phenotypic effect or evolutionary conservation rates, as well as gene features such as haploinsufficiency and essentiality predictors. In addition, beyond the estimation of the proportion of patients under a monogenic model, we also propose exploring other possible disease models such us the oligogenic or polygenic by considering the presence of mutations in interacting proteins or the accumulation of functional variants in immunological pathways, as well as the disease association with rare functional genetic variants by comparison to healthy controls (17).

## Materials and Methods

### Individuals Included in the Study

This study includes 36 patients diagnosed with CVID, including both sporadic and familiar cases, without any genetically confirmed primary immunodeficiency (PID), and completing the conventional criteria for CVID classification: (1) from 2 to 18 years old at the age of diagnosis; (2) lack of antibody production after immunization of antigen exposure in at least two assays; (3) 2 years post-diagnosis to exclude lymphoid malignancy; (4) IgG levels 2.5th centile for age and low IgA or/and IgM levels. CVID patients presenting one of the following features were excluded from the study: (a) well-known gene-identified PID such as hyper IgM; CD19+ or CD20+ B cell deficiency; ICOS or transmembrane activator and calcium-modulating cyclophilin ligand interactor (TACI) gene mutation already diagnosed; (b) secondary immunodeficiencies such as those due to complications such as associated tumors and lymphomas or from other therapies (side-effects following splenectomy, corticosteroid, or immune suppressive therapies). Patients L283, L286, and N216 were reported to be consanguineous. In addition, parents and siblings have also been included in the study, when available. Written informed consent for genetic analysis and research was obtained from all participants and ethical approval for the project was obtained from the institutional ethical committees.

We used two different sets of controls: whole-exome sequences from 36 individuals from a Spanish cohort diagnosed with autism spectrum disorders (ASD) (18) and 267 whole-exome sequences from healthy controls from a Spanish cohort (19). In the case where no data were available for the 267 whole-exome sequences, we retrieved data from the CIBERER Spanish Variant Server (csvs.babelomics.org) and used data for individuals with different syndromes not related to primary immunodeficiencies.

### Genetic Analyses

DNA was extracted from blood samples. CNV analysis was performed with the CytoScanHD array (Affymetrix) according to the manufacturer’s protocol. The CytoScanHD array contains 743,304 SNPs and 2,696,550 CNV markers. The obtained cychp files were analyzed with Chromosome Analysis Suite v.2.1.0.16 software and NetAffx na33 annotation version. For CNV detection and to prevent false positives, we considered alterations involving at least 25 markers and more than 150 Kb in length for gains, and 35 markers and more than 75 Kb for losses. For detection of loss of heterozygosity (LOH) regions, we considered alterations of at least 50 markers in more than 5 Mb. Exome capture was performed with the Agilent SureSelect XT enrichment system. DNA was sequenced in an Illumina HiSeq 2000 platform in a 2 × 75 paired-end cycles run. PCR duplicates were removed with Picard.^2^ Sequence reads were mapped to the human reference genome (hg19) using GEM (20). Variant calling was performed using GATK (21) and SNP annotation with SnpEff (22) and SnpSift (23). Candidate mutations were visually inspected with the Integrative Genomics Viewer (24) and, when required, validated by Sanger sequencing. Somatic variants analysis was performed with VarScan2 (25), considering the high impact variants predicted with SnpEff (22), *P*-value <0.05, present in less than 40% of the reads and in a maximum of two patients.

### Genetic Data and Statistical Analyses

Only functional variants were considered, including missense, stop-gain and stop-loss, splice donor or acceptor sites mutations, and frameshift insertions and deletions. In addition to standard filters for mapping and variant calling and annotation we also discarded indels clustering within 10 base pairs of another indel and for most of the analyses we excluded those variants present in 10 or more individuals in our dataset. We used allele frequencies from The 1000 Genomes Project (26) and the NHLBI and Exome Sequencing Project.^3^ We used GERP (27, 28) to asses for evolutionary conservation and Polyphen (29) and SIFT (30) to predict the phenotypic impact of missense variants. We have also used predicted haploinsufficiency (31), intolerance to functional variation (32), and essentiality (33) scores to infer the possible model of the disease and prioritize candidate genes in the different patients.

We used the Fisher’s exact test to assess the statistical significance of an excess of rare functional variants in cases compared to controls, from two by two tables with the total number of rare functional variants, and the total number of synonymous variants in patients and controls. In both cases, variants present in more than 10 individuals were excluded from the analysis to exclude false positives produced by sequencing artifacts. We applied the Li and Leal’s collapsing method (34) to detect an excess of CVID patients with rare functional variation when compared to controls. Statistical significance was also assessed using the Fisher’s exact test. For these two analyses, only nucleotide substitutions were considered.

The protein–protein interaction (PPI) data was obtained from the Human Protein Reference Database (35) considering the whole set of non-redundant interactions between two proteins. Gene lists for each pathway were extracted from the KEGG database (36–38). We considered the 25 pathways shown in Table S1 in Supplementary Material.

### Functional Validations

To assess the effect of specific gene alterations, additional functional tests were performed. Mainly with peripheral blood mononuclear cells (PBMCs) or Epstein–Barr transformed B cells (EBV-B), including lymphocyte phenotyping and western-blot Ficoll–Hypaque (Sigma-Aldrich, St. Louis, MO, USA) density gradient centrifugation of heparinized blood was used for PBMC isolation. Cells were cultured with complete medium [RPMI (Gibco, Grand Island, NY, USA) supplemented with 10% heat-inactivated fetal calf serum (Sigma-Aldrich, St. Louis, MO, USA), 1 µg/ml penicillin and 1 µg/ml streptomycin (Invitrogen, Grand Island, NY, USA)]. Viable cells were counted using a hemocytometer in an inverted microscope.

CTLA-4 expression detection was performed as described elsewhere (39–42). Specifically, for Treg cell phenotyping and CTLA-4 expression PBMCs were left with medium (resting) or stimulated with PHA (5μg/ml, Sigma-Aldrich, St. Louis, MO, USA) for 24 h. Treg intracellular staining was performed with Treg Detection Kit (CD4/CD25/FoxP3) kit (Miltenyi Biotec, Germany) following manufacturer’s instructions. CD3 BV421 (BD bioscience, San Jose, CA, USA), CD4 FITC, and CD25 APC (Miltenyi) were used for extracellular staining and FoxP3 APC (Miltenyi) and CTLA4 PE (BD biosciences) for intracellular staining and then acquired with the cytometer (FACS Canto II, BD biosciences).

Lymphocyte stimulation capacity was assessed by flow cytometric detection of activation markers. PBMCs were stimulated for 7 days and then surface-stained with the following antibodies against activation markers: CD62L, CD25, HLA-DR, CD69, and CD40-L (BD Biosciences) and then acquired with the cytometer (FACS Canto II, BD biosciences).

Protein extraction and Western Blot: LRBA determination was performed in EBV-B cells (43). EBV-B cells were lysed with 1% NP-40 buffer. Protein concentration was normalized between control and patient. Products were analyzed by sodium dodecyl sulfate-polyacrylamide gel electrophoresis and western blotting. A nitrocellulose membrane was blocked with a 2% milk TBS, then incubated overnight with primary antibodies anti-LRBA (1:500, polyclonal, Abcam, United Kingdom) and anti-GAPDH (1:1000, polyclonal, Bio-Rad, United Kingdom) then the membrane was washed with TTBS and incubated for 1,5 h with Goat Anti-Rabbit IgG H&L (HRP) (1:5000, Abcam). It was then developed with SuperSignal™ West Pico Chemiluminescent Substrate (Thermo Scientific, Waltham, MA, USA) following the manufacturer’s instructions and acquired with ImageQuant LAS-4000 (GE Healthcare Life Sciences, Buckinghamshire, England, UK) equipment.

## Results

We generated whole-exome sequencing data for the 36 CVID patients included in the study, as well as for eight relatives, with an average coverage of 120×. In addition, we also generated CNV data for all the samples except in one case where DNA was not available. Table S2 in Supplementary Material shows the number of functional genetic variants described in each sample, classified in different annotation categories: missense, stop-gain (or nonsense), start-gain, splice site, and inframe and frameshift indels, with total numbers similar to what has been previously reported (44). Table S2 in Supplementary Material also contains the number of structural variants and LOH regions detected in the genotyping analysis with the CytoScanHD array.

### OMIM CVID-Causing Mutations

The OMIM database^4^ includes known variants originating CVID in 13 genes: *ICOS, TNFRSF13B, TNFRSF13C, CD19, CR2, MS4A1, CD81, IL21, LRBA, NFKB1, NFKB2, PRKCD*, and *IKZF1*. There is also evidence that defects in other genes (*CTLA4, PLCG2*) can cause a similar phenotype or modify the severity of the disease with comorbidities (*MSH5*). These genes are mainly related to T-cell and B-cell defects leading to a deficiency in antibody production. In these 16 genes, we found a total of 96 nucleotide variants and 6 CNVs previously described to be putatively related to CVID in the literature (Table S3 in Supplementary Material). Four of them were found in the CVID patients of this study (Table 1).

**Table 1:** Known common variable immunodeficiency (CVID) variants detected in CVID patients in this study.

Gene	cDNA	Aa change	Genotype^a^ (reference)	hg19_pos	CVID (*N* = 36)^b^	Controls (literature)	Controls (Autism, *N* = 36)^b^	Controls (Spain, *N* = 267)^b^
*TNFRSF13B*	c.752C > T	p.P251L	0/1 (10)	17:16842991	9	Yes	0	36 (3)
*TNFRSF13B*	c.310T > C	p.C104R	*/1 (45)	17:16852187	3 (1)	Yes	0	2
*TNFRSF13C*	c.62G > C	p.P21R	2*0/1 (46)	22:42322716	4	Yes	0	16^c^
*MSH5*	c.253C > T	p.L85F	2*0/1 (47)	6:31709045	2	Yes	0	55 (2)

^a^0/1 heterozygotes; 1/1 homozygotes; */1 heterozygotes and homozygotes; 2*0/1 compound heterozygotes.

*^b^Homozygous individuals are shown in brackets*.

*^c^No data available for the 267 controls. Instead, we used data from 578 whole-exome sequences at the CIBERER Spanish Variant Server (csvs.babelomics.org)*.

Two of the reported variants are included in the *TNFRSF13B* gene (TACI), which is known to harbor functional mutations in 5–10% of patients diagnosed with CVID (48, 49). However, the existence of healthy controls with heterozygous mutations in this gene and the lack of a clear Mendelian pattern of inheritance in families have led to consider some of the mutations at *TNFRSF13B* as risk factors (9, 10) which could be determinant only in the case of homozygous individuals (50). Thus, *TNFRSF13B* would be considered a modifier gene rather than a causal gene in monogenic cases (51). The p.C104R variant is the most common *TNFRSF13B* functional mutation found in CVID patients (51). Three of the patients in this study present this mutation, in one case in homozygous state, being the second case found to date (52). This mutation is significantly more frequent in our CVID patients compared to the Spanish cohort controls (19) (*P* = 0.003, Fisher’s exact test) and absent in the ASD controls (18) (Table 1). In the same gene, we report nine samples with the protein change P251L, although in this case the proportion is not significantly higher than in controls. In addition, a direct causal role for this variant can probably be discarded because of its high frequency in the reference populations (14% in the ExAc database, 11% for the European population). On the other hand the P21R variant of the *TNFRSF13C* gene found in four patients, and also one healthy parent, shows a higher frequency when compared to controls (*P* = 0.003, Fisher’s exact test). However, this variant (rs77874543) has also been found in non-CVID exomes in homozygosity, and has a population frequency higher than 5%. Finally, we also detected two patients with the L85F substitution in the *MSH5* gene (47). The same aminoacid substitution was also present in the mother of one these patients, not diagnosed with CVID but with some of the clinical features described in the patient. Nonetheless, this genetic variant has been found at lower frequencies in CVID patients compared to controls, and has a population frequency of 2% or higher in some populations (7% in Africans), which suggests that it does not have a determinant role in CVID.

### Loss-of-Function (LoF) Variants

Loss-of-Function variants include stop-gain and loss mutations, splice-site mutations, and frameshift indels, which are predicted to disrupt proteins and, therefore, could likely relate to disease phenotypes, and in fact account for approximately 20% of the coding variants associated with disease (53). Table S2 in Supplementary Material shows the number of LoF variants identified in each individual of the study. The number of LoF variants ranges from 78 to 153, similar to what has been previously described (44, 53, 54). Applying different frequency thresholds substantially reduces the number of LoF variants per individual (54, 55). We established a permissive allele frequency threshold of 1%, and first focused the analysis on the LoF variants described in candidate genes for CVID (Table 2). With this aim, we generated a list of 97 candidate genes for CVID (Table S4 in Supplementary Material), including genes in the OMIM database,^5^ genes defined in a review by Bogaert and colleagues (51), and others from the literature. Second, we also analyzed the presence of LoF variants in proteins interacting with the proteins encoded by candidate genes (see Materials and Methods) (Table 2). Finally, we also report all the genes with LoF variants using a very low frequency threshold (0.001) (Table S5 in Supplementary material).

**Table 2:** Genes with Loss-of-Function (LoF) homozygous or heterozygous variants in common variable immunodeficiency (CVID) candidate genes and interacting proteins.

Individual	CVID < 0.01	PPI < 0.01
L283	*LRBA*(hom)	
L287		*C7orf64*(het), *PDGFRB*(hom), *RIPK4*(het)
L289		*HDAC1*(het)
L291		*GP6*(het)
L292	*NOD2*(het)	*SLA2*(het), *ZNF655*(het)
L297	*NFKB1*(het)	
L298		*MAPK8*(het)
L299		*FGFR3*(het)
N202	PIK3R1(het)	FHOD1(het)
N204		*HP*(het), *PLSCR1*(het)
N205		*HNF1A* (comp_het)
N206		*R PA 2*(het)
N207	*NFKB1*(het)	
N208		*EEF1G*(het)
N210		*DERL3*(het), *HP*(het), *PDGFRB*(het)
N211	*CTLA4*(het)	
N213		*IBTK*(het), *PDGFRB*(het)
N216		*CASP1*(het), *HCLS1*(het), *NCOR2*(het)
N223		*BCAP31*(het), *SLC6A8*(het), *TNFRSF12A*(het)
N224		*BCAP31*(hom), *CASP1*(het), *SLC6A8*(hom), *TNFRSF12A*(het)
N227		*BCAP31*(het), *SLC6A8*(het)
N229		*CR1*(het), *SPI1*(het)
N231		*PML*(het), *TNFRSF12A*(het)
N232		*TNFRSF12A*(het)
N233	*NOD2*(het)	*TNFRSF12A*(het)
N234	*IL10RA*(het), *NFKB1*(het)	*TNFRSF12A*(het)
N235		*C9*(het), *PIAS1*(het), *TRPV1*(het)

*PPI, protein–protein interaction*.

**Figure 1:**
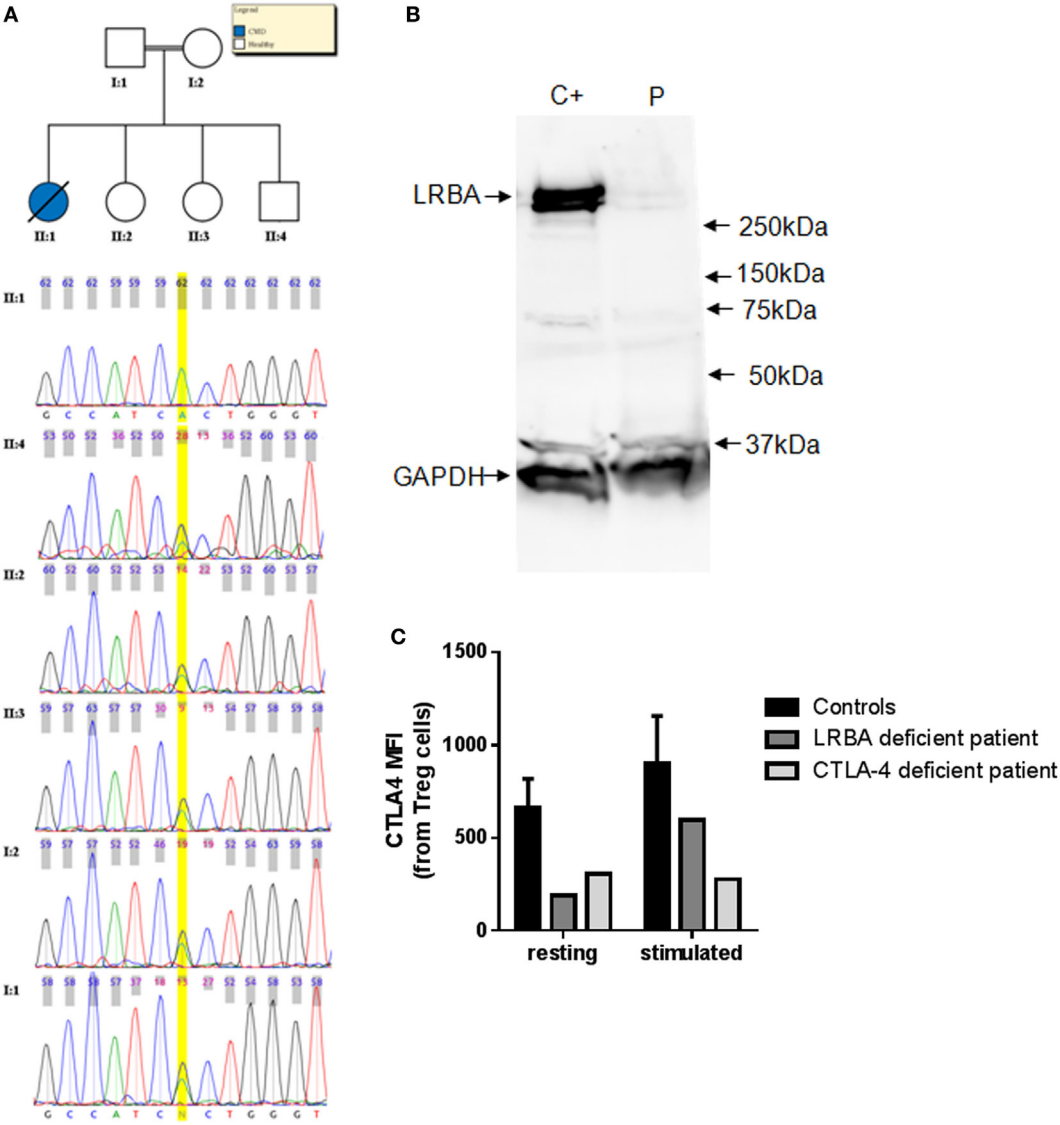
**(A)** Chromatograms corresponding to the Sanger sequencing of the LRBA nonsense mutation region in L283 and five healthy relatives. **(B)** Western blot analysis of LRBA and GAPDH for L283 patient (P) and a healthy control C+. LRBA protein is not detectable in the LRBA-deficient patient. **(C)** CTLA4 expression is downregulated in LRBA- and CTLA4-deficient patients. CTLA4 expression was assessed in Treg cells (CD3+CD4+CD25hiFoxP3+ cells) in resting and in PHA-stimulated cells (24 h). Bars represent mean values and error bars represent SE of the mean values for adult healthy controls (*n* = 5).

Eight patients harbor a LoF variant at a frequency less than 1% in CVID candidate genes (Table 2). Among them, L283 presents a new homozygous nonsense variant at the exon 4 of the *LRBA* gene [chr4:151392836G > A (hg19)]. This stop codon at *LRBA* (R2214*) is introduced at the beginning of the BEACH domain (IPR000409 in InterPro), a highly conserved domain with known crystal structure but unknown function (56). This mutation was validated by Sanger sequencing in the patient, and also detected in heterozygosis in both parents and three healthy siblings (Figure 1A). Copy number and SNP analyses confirmed the existence of consanguinity in this patient. We estimated a consanguinity index of 0.058 compatible with descendants from third degree kinship marriages, based in the total of 174 Mb included in LOH regions (57), with 10 LOH regions of more than 5 Mb. We then performed assays with the patient cells to test the effect of the variant on the protein. The western blot gel electrophoresis separation (Figure 1B) shows that the cells of the patient do not produce any detectable amount of LRBA protein, thus validating the deleterious effect of the mutation abolishing protein production probably through nonsense-mediated decay. Furthermore, the expression of CTLA4 is downregulated in Treg cells of the LRBA-deficient patient (Figure 1C), in agreement with the previous description of CTLA4 detection in Treg cells from LRBA-deficient patients (39).

**Figure 2:**
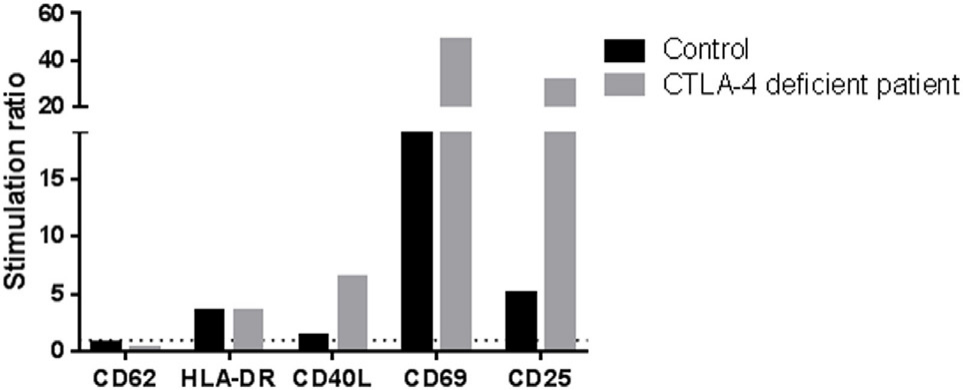
Stimulation ratio of different lymphocyte activation markers after PHA stimulation (7 days). Stimulation ratio: mean fluorescence intensity of PHA-stimulated/basal conditions.

The N211 patient presents a new LoF genetic variant located at the *CTLA4* gene, which has already been reported to harbor causal heterozygous CVID variants (41, 42). The mutation causes a frameshift deletion not previously described and absent in the reference databases. We performed Sanger sequencing of this mutation and confirmed that it is a *de novo* mutation absent in the parents (Figure S1 in Supplementary Material) and, therefore, a strong candidate to originate CVID. We performed functional analyses to study the expression of *CTLA4* in Treg cells and we found that it is downregulated before and after stimulation with PHA. CTLA4 detection was lower than in the case of the aforementioned LRBA-deficient patient after PHA stimulation (Figure 1C). Finally, we also analyzed the lymphocyte stimulation in the patient. After 7 days stimulation with PHA, the stimulation ratio of different lymphocyte stimulation markers was increased in the patient compared to a healthy control (Figure 2).

N202 presents a heterozygous splicing variant in PIK3R1. This variant has been previously reported to originate an immunodeficiency because of its dominant gain of function effect on PI3K signaling (58) in agreement with its high haploinsufficiency prediction value of 0.89 (31). For the remaining five patients presenting a low frequency heterozygous LoF variant in a CVID candidate gene (Table 2), three of them have a variant in *NFKB1*, which has also been reported to harbor heterozygous mutations originating CVID (7). Two of them share a start loss variant affecting one of the transcripts, although its frequency of 0.002 makes it unlikely to have a causal (monogenic) role in the disease. By contrast, a new splice-site mutation in *NFKB1* is described in N234, being a good candidate to originate the disease. In addition, N227 presents a 13 MB heterozygous deletion (chr4: 94,135,868–107,295,574) not present in parents which includes the *NFKB1* gene among others (Table S2 in Supplementary Material). Finally, although the variants described at *NOD2* and *IL10RA* are not present in any database, no CVID cases with heterozygous variants at these genes have been described, in agreement with their low haploinsufficiency values (0.119 and 0.173, respectively). In addition, Table 2 also includes low frequency LoF variants of genes interacting with candidate genes related with CVID.

### Functional Genetic Variation at Candidate Genes for CVID

We then explored the presence of functional variants, other than LoF described above, in candidate genes for CVID. The final number of variants with frequency less than 1% in each individual is shown in Table S6 in Supplementary Material, differentiating variants in candidate genes, variants in interacting proteins and in other genes. We excluded from this, and subsequent analyses, the two individuals with a functionally validated LoF candidate (L283 and N211, see above), and the variants also present in healthy relatives (when this information is available from exome sequencing). We first analyzed the presence of single variants in CVID genes that could originate the disease following a dominant model. Because the number of genes with one or more functional variants is too high we applied stringent filters to produce a short list of candidate genes. We selected the variants with a GERP conservation score higher than 2 (28), a Polyphen score higher than 0.5 (for nucleotide variants) and a frequency in the ExAC and GMAF databases below 0.001. The nine variants at CVID genes fulfilling these conditions are shown in Table 3. Two of them are in frame indels and, therefore, less prone to have an effect on the protein. Among the heterozygous missense variants, *PRKCD, CLEC16A*, and *DOCK8* (this latter absent in the healthy sister N209) are the more interesting candidates, considering their haploinsufficiency predictions (31) and essentiality values estimated from network and evolutionary properties (59). We then considered the recessive genetic model with the disease being originated by two rare functional variants in the same gene. We analyzed the presence of homozygous variants or compound heterozygotes in CVID candidate genes, at frequencies below 0.01 (Table 4). Interestingly, two candidate genes (*CR2* and *PLCG2*) are found as compound heterozygotes in patients N233 and N212, respectively.

**Table 3:** Functional heterozygous genetic variants with high predicted phenotypic effect at common variable immunodeficiency (CVID) candidate genes.

Patient	chr	Position	Gene	Function	Polyphen	rs	GERP	esp5400_all	HI1	HI2	RVIS	Essent
L294	chr9	100,774,719	*ANP32B*	Inframe indel	–	–	–	0	0.808	0.655	–0.16 (41.25%)	0.89
N214	chr7	2,976,742	*CARD11*	Missense	0.654	–	2.18	0	0.181	0.517	–1.39 (4.33%)	0.81
N212	chr11	60,892,540	*CD5*	Missense	0.936	–	3.08	0	0.284	0.402	0.8 (87.66%)	0.666
N232	chr1	160,523,750	*CD84*	Missense	0.999	rs146076557	5.25	0.000279	0.132	0.488	0.04 (57.15%)	0.077
L292	chr16	11,073,195	*CLEC16A*	Missense	0.857	rs74163607	5.3	0.000201	NA	0.578	–1.01 (8.2%)	0.547
N201	chr1	207,651,294	*CR2*	Missense	0.659	rs146465618	5.59	0.000093	0.234	NA	0.06 (57.56%)	0.558
N210	chr9	377,046	*DOCK8*	Missense	0.868	rs148693111	5.71	0.000186	0.535	0.57	–1.94 (1.9%)	0.845
L287	chr1	234,744,945	*IRF2BP2*	Inframe indel	–	–	2.62	0	0.852	0.626	–	0.992
N213	chr1	234,744,945	*IRF2BP2*	Inframe indel	–	–	2.62	0	0.852	0.626	–	0.992
N216	chr1	234,744,945	*IRF2BP2*	Inframe indel	–	–	2.62	0	0.852	0.626	–	0.992
L288	chr3	53,218,928	*PRKCD*	Missense	0.733	–	5.91	0	0.636	0.553	–1.04 (7.77%)	0.966

*HI1 and HI2 haploinsufficiency predictions (24); RVIS, residual variation score of genetic variation intolerance (25) with the percentile of intolerant human genes in parentheses; Essent, essentiality index estimated from network and evolutionary properties (43)*.

**Table 4:** Compound heterozygotes at common variable immunodeficiency (CVID) genes.

chr	Position	ref	alt	Polyphen	rs	GERP	esp5400 all	GMAF	Effect	Gene	idsample	Genotype
chr1	207643100	C	A	0.002	–	−3.25	–	–	Non-synonymous	*CR2*	N233	0/1
chr1	207648456	G	T	0.05	rs144572703	4.47	0.005763	0.0018	Non-synonymous	*CR2*	N233	0/1
chr16	81939089	T	C	0.598	rs187956469	5.18	0.002838	0.0032	Non-synonymous	*PLCG2*	N212	0/1
chr16	81942175	A	G	0.005	rs75472618	6.5	0.007067	0.0064	Non-synonymous	*PLCG2*	N212	0/1

### Compound Heterozygotes at Non-CVID Genes

We expanded the analysis beyond the list of CVID candidate genes to the rest of the genome. We based our approach on the use of stringent filters (frequency, conservation, predicted effect) and the consideration of predictors of the degree of essentiality of the gene. This approach produces a list of new candidate genes in each CVID patient which can be ranked using the different variant and gene properties. We produced a list of genes harboring compound heterozygotes in each patient and applied two different allele frequency thresholds of 0.01 and 0.001. Table 5 shows the number of compound heterozygotes per patient, and gene names are shown in Table S7 in Supplementary Material. The number of genes per patient can be reduced using additional filters based in evolutionary conservation or predicted phenotypic effect. We established a threshold of a GERP > 2 for the functional variants, since positions with values greater than 2 are considered to be conserved among mammals and, therefore, more to prone to be of functional importance (28). On the functional effect, we used the Polyphen prediction and established a threshold value of 0.5 (60) (Table 5). Table S8 in Supplementary Material also shows additional information on gene properties which might aid the prioritization of candidate genes. Four genes are detected as compound heterozygotes in more than one patient (with GERP > 2 and Polyphen > 0.5): *SLC25A5* (eight), *ACOT4* (7), *KMT2C* (two), and OR10X1 (two). However, *SLC25A5* and OR10X1 are two genes which have been recurrently reported in next-generation sequencing studies (61), probably because of being prone to mapping artifacts and, thus, to accumulating false variants. On the other hand, *ACOT4* (with a function apparently not related to the immune function), is also a paralog of *ACOT1*. Finally, *KMT2C* is also present in two patients, although one of them is N227 which harbors a large deletion encompassing *NFKB1* among other genes.

**Table 5:** Number of genes harboring compound heterozygotes mutations in the patients included in this study.

Sample	Genes 1%	Genes 0.1%	Genes 1% filtered^a^	Genes 0.1% filtered^a^
L283	53	21	*FAM186B, MYH11, SLC25A5, SDK1*	*FAM186B*
L287	26	11	*CBS, TRIB3*	0
L288	29	18	0	0
L289	39	19	*FHL3, SLC25A5, FMN2*	0
L290	31	15	0	0
L291	27	18	*CAMPSAP3, VPS13C*	*CAMPSAP3*
L292	26	16	*PKHD1L1, PLEC, MLH1*	*PKHD1L1*
L294	31	20	*PRSS16*	*PRSS16*
L295	34	15	0	0
L296	25	13	0	0
L297	30	17	*SLC25A5*	0
L298	28	7	*BMP1*	0
L299	27	14	*ACOT4, GPR112, SLC25A5, UNC13C*	*ACOT4, GPR112*
N201	33	23	*ACOT4, SLC25A5, Z FYVE26*	*ACOT4*
N202	27	13	*ACOT4, WFS1, SLC25A5*	*ACOT4*
N203	33	13	*SLC25A5, SEPT1*	0
N204	34	14	*ACOT4*	*ACOT4*
N205	28	15	0	0
N206	34	18	*ACOT4*	*ACOT4*
N207	29	17	0	0
N208	23	12	0	0
N210	33	18	*EPPK1*	0
N211	25	13	*GLTSCR1*	0
N212	25	11	*ACOT4*	*ACOT4*
N213	27	10	*ACOT4, SLCS5A5*	*ACOT4*
N214	19	6	*SLC25A5*	0
N216	75	21	*PENK, NUP214, KDM4C*	*PENK*
N223	35	19	*OR10X1*	0
N224	38	22	*OR10X1*	0
N227	37	25	*KMT2C*	0
N229	39	20	0	0
N231	35	20	*BAI1, TTN, MTDH*	*BAI1*
N232	18	12	*KMT2C, PMFBP1*	0
N233	35	19	0	0
N234	37	22	*CMYA5*	0
N235	42	22	0	0

*^a^Variants with GERP > 2 and Polyphen > 0.5*.

### Oligogenic Disease

For the patients without a clear candidate gene for a monogenic origin of the disease, we then considered an oligogenic model of inheritance. In particular, we considered the digenic model. DIDA, a database of digenic diseases, included 44 diseases with 213 digenic combinations collected from the literature until June 2015 (62). This form of disease refers to both situations with a primary and a secondary locus or cases where two loci contribute to the disease with roughly the same importance (63). Modifier genes, affecting the severity of the disease, can also be considered a type of digenic inheritance (64).

The case of *TNFRSF13B*, with several common variants related to CVID but with reported healthy carriers, could fit with this digenic model where additional variants would be needed to develop the disease. We analyzed the two patients with variants in this gene (Table 1), and searched for variants in genes interacting with *TNFRSF13B*. Patient L297 harboring the C104R change in homozygosis, also has a heterozygous missense variant with a 2% frequency in *TNFRSF13C*, which directly interacts with *TNFRSF13B*. No other variants in interacting proteins were described in the patients with known CVID variants in *TNFRSF13B, TNFRSF13C*, or *MSH5* (Table 1). We expanded this analysis by assessing the presence of heterozygous rare functional variants in a CVID gene and in an interacting protein in the same patient. Table 6 shows the 10 patients in which this situation has been found, considering variants with GERP > 2 and below 0.01 frequency (see Table S9 in Supplementary Material) when considering a maximum frequency of 0.05. Interestingly, two pairs of related patients (sisters N205 and N206, and brothers N207 and N208) share the presence of variants at the interacting proteins PIK3R1-AXL and PIK3CD-RALY, respectively. In four more patients (Table 6), the CVID genes had already been suggested as probably causal (Tables 2–4) following recessive (N233) or dominant models (L288, N210, N234).

**Table 6:** Patients with rare functional variants (MAF < 0.01) and GERP > 2 in a common variable immunodeficiency (CVID) candidate gene and interacting proteins.

Patient	CVID gene	Variants	Interacting protein	Variants
L288	*PRKCD*	1 het	*RUNX2*	1 hom
L293	*DOCK8*	1 het	*CDC42*	1 het
L299	*STAT1*	1 het	*FGFR3*	1 hom
L299	*STAT1*	1 het	*FGFR4*	1 hom, 1 het
N205	*PIK3R1*	1 het	*AXL*	1 hom
N205	*PIK3R1*	1 het	*TYK2*	1 het
N206	*PIK3R1*	1 het	*AXL*	1 hom
N207	*PIK3CD*	1 het	*RALY*	1 het
N208	*PIK3CD*	1 het	*RALY*	1 hom
N210	*DOCK8*	1 het	*CDC42*	1 het
N233	*CR2*	1 het	*FHOD1*	1 het
N234	*RAD50*	1 het	*NBN*	1 het
N234	*NFKB1*	1 het	*NCOR2*	2 het

**Figure 3:**
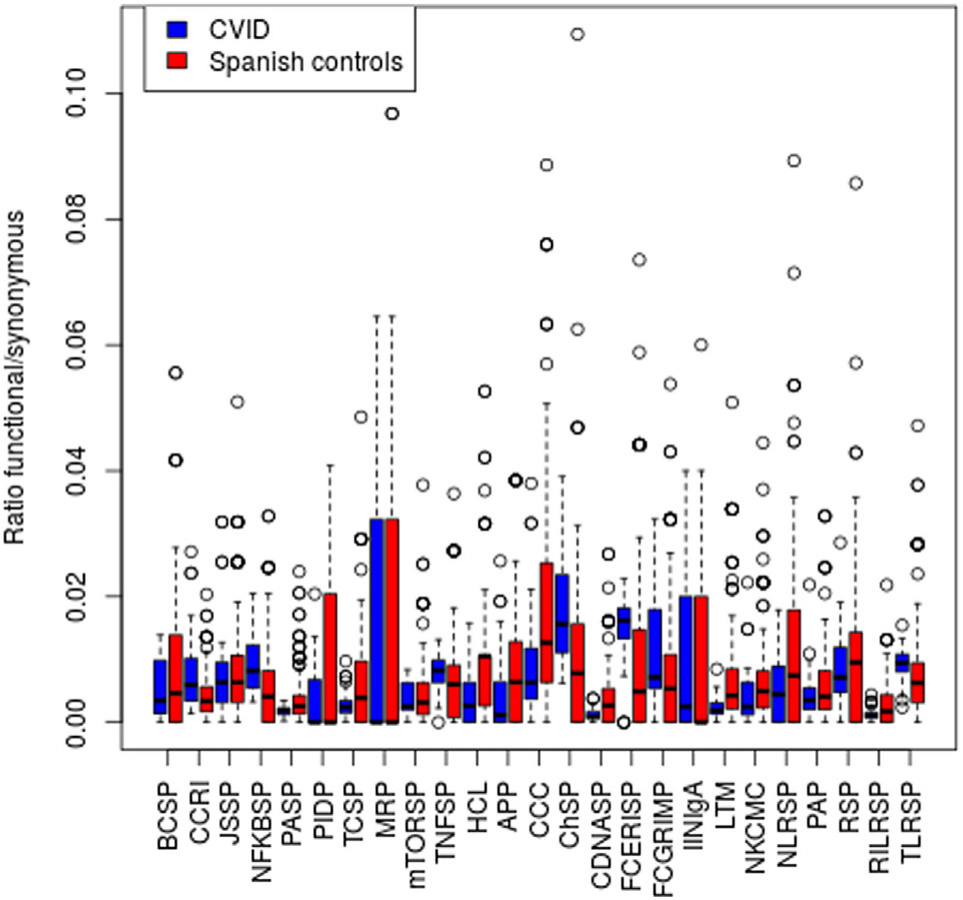
Number of functional genetic variants in common variable immunodeficiency (CVID) patients and controls in immunological pathways. Abbreviations and the number of genes in each pathway are shown in the Section “Materials and Methods.”

We then expanded the analysis to a scenario where variants in several genes of an individual might contribute to the disease. For this purpose, we assessed the presence of particular CVID patients which compared to the rest of the patients in the study harbors an excess of rare functional variants at any of 25 KEGG pathways related to the immune function (36) (Figure 3). We used a frequency threshold of 1% and estimated the ratios of functional to synonymous variants in each sample, to correct for possible differences in coverage across samples. We considered as outliers those individuals departing from twice the SD of the average number of rare functional variants (Figure 3). Table 7 shows the CVID patients with an excess of rare functional genetic variants in a particular pathway. The presence of more than one pathway in three of the patients is mostly due to the fact that these patients have genetic variants in genes with a role in several pathways. In the case of patient N208, it shows an excess of variants in five pathways that share the presence of three MAP Kinases (*MAPK14, MAP2K2*, and *MAP2K3*). Of interest, we found four patients with an excess of rare functional genetic variants in the B cell signaling pathway and three in the T cell signaling pathway, in addition to another two in the tumor necrosis factor and Fc epsilon RI signaling pathways (Table 7).

**Table 7:** Pathways with an excess of genes with rare functional variants in common variable immunodeficiency (CVID) patients.

Patient	Pathway	Gene^a^
L296	BCSP	*CD79A, CR2, DAPP1, PLCG2*
L297	TNFSP	*CREB3, CREB3L1, NFKB1, PIK3CG, TNFRSF13B*
N207	BCSP	*PIK3CD, DAPP1, NFKB1, CD81, NFATC3*
	TCSP	*PIK3CD, NFKB1, MAP14, NFATC3*
N208	BCSP	*PIK3CD, IKBKB, CD81, NFATC3, MAP2K2*
	FCERISP	*PIK3CD, MAPK14, MAP2K3, MPA2K2*
	TCSP	*PIK3CD, MAPK14, IKBKB, NFATC3, MAP2K2*
	TLRS	*PIK3CD, MAPK14, IKBKB, IFNA14, MAP2K2, MAP2K3*
	TNFSP	*PIK3CD, MAPK14, IKBKB, MAP2K3*
N210	TCSP	*CDC42, MAP3K8, PAK6, SOS2*
N212	BCSP	*PIK3CG, PLCG2*(2), *NFATC2, MAPK1, RAC2*
	FCERISP	*PIK3CG, PLCG2*(2), *MAPK1, RAC2*
	NKCMC	*PIK3CG, PTK2B, PTPN11, PLCG2*(2), *NFATC2, IFNAR1, MAPK1, RAC2*
	NLRSP	*CASP5, NLRP1*(4), *MAPK1*
N216	MRP	*MLH3, MSH2, PMS2*
N229	PAP	*PRKCZ, GUCY1B3, LCP2, ITPR2, ORAI1, PLA2G4B, ROCK1, TBXA2R, PIK3R2*
N231	IINIgA	*HLA-DRB1*(2), *TNFRSF13B, ICOSLG*

*^a^Number of genetic variants at this gene when is greater than one, indicated in brackets*.

### Association to Rare Variants

Next, we assessed the association of rare functional genetic variation to CVID. In this case, analyses are performed to detect an excess of rare functional variation in a particular gene or pathway in CVID patients compared to controls, rather than the detection of the causal genetic variant(s) in particular individuals. To analyze the presence of genes harboring an excess of rare functional variants in the CVID patients compared to healthy controls, we first compared the ratio of rare functional to synonymous variants for each gene in cases compared to healthy controls. Table 8 shows the results of the analysis for the 60 genes analyzed (with at least one synonymous variant in each cohort), for the 34 patients without a validated candidate gene for a monogenic origin of the disease. Four genes (*PRKCD, CLEC16A, DOCK8*, and *PLCG2*) show a statistically significant excess of rare functional variants in CVID patients, after applying Bonferroni’s multiple test correction. Table S10 in Supplementary Material reports these functional variants and their properties.

**Table 8:** Excess of rare functional variants in common variable immunodeficiency (CVID) patients.

Gene	Rare Funct CVID	Syn CVID	Rare Funct Controls	Syn Controls	*P*-value
*PRKCD*	3	4	5	1693	2.36e−06
*CLEC16A*	6	11	8	321	1.30e−05
*DOCK8*	3	11	0	347	4.68e−05
*PLCG2*	4	14	18	1465	9.28e−05

*Rare Funct, number of rare genetic variants; Syn, number of synonymous variants*.

Second, we used the Li and Leal’s collapsing method (34) to detect an excess of CVID patients harboring rare functional genetic variants. In this method, individuals with and without at least one functional rare variant are compared between CVID patients and controls. This test has been performed only for those genes with similar lengths for the targeted regions to avoid false positives with more functional variants because of a larger scanned region in CVID patients. Table 9 presents six genes (*PIK3CD, ICOSLG, TNFRSF13B, PIK3R1, CD84*, and *PRKCD*) showing a statistically significant excess of individuals with rare functional variants in CVID cases when compared to controls. Interestingly, *PRKCD* showed also a significant excess of functional variation in cases in the previous analysis (Table 8), although only *PIK3CD* remains significant after Bonferroni’s correction. Genetic variants in each gene are shown in Table S10 in Supplementary Material.

**Table 9:** Common variable immunodeficiency (CVID) genes with an excess of patients harboring rare functional genetic variants in patients compared to controls.

Gene	Patients funct^a^	Patients no funct^b^	Controls funct^c^	Controls no funct^d^	*P*-value
*PIK3CD*	9	25	3	264	1.84E−07
*ICOSLG*	4	30	6	261	0.018
*TNFRSF13B*	3	31	4	263	0.034
*PIK3R1*	2	32	1	266	0.035
*CD84*	3	31	5	262	0.050
*PRKCD*	3	31	5	262	0.050

*^a^Number of patients with at least one rare functional genetic variant*.

*^b^Number of patients with no rare functional genetic variants*.

*^c^Number of controls with at least one rare functional genetic variant*.

*^d^Number of controls with no rare functional genetic variants*.

Finally, we assessed a possible excess of functional variants in the 25 KEGG pathways by comparing our CVID patients to a set of controls (see Materials and Methods), by comparing the ratios of rare (<1%) functional to synonymous variants in each sample. We detected a significant excess of variants in two of the pathways in CVID patients when compared to controls: Fc epsilon RI signaling and cytokine–cytokine receptor interaction pathways (*P* < 0.001 and 0.002, respectively), plus two other marginally significant pathways after applying multiple test correction: cytosolic-DNA sensing, and NFKB signaling (*P* = 0.002 and 0.001, respectively). The four pathways also show a significant excess of functional variation in CVID patients when compared to the ASD controls set.

## Discussion

**Figure 4:**
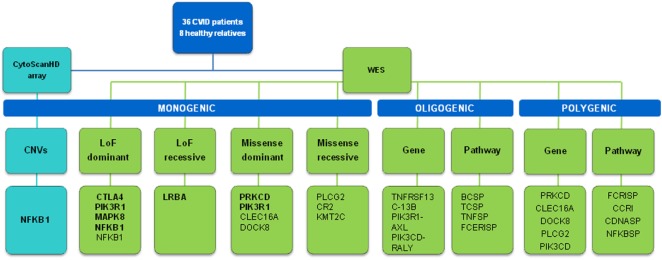
Scheme of the approaches and main results of this study. Candidate genes in bold are those with more evidences of being causal.

In this work, we first approach the proportion of monogenic cases in CVID by using deep whole-exome sequencing combined with CNV analysis, in a cohort with mostly early diagnosis patients (and all of them less than 18 years old), which is expected to optimize the probability of including monogenic cases (15). We propose candidate genetic variants and genes with different levels of confidence (Figure 4). The higher confidence cases are the five LoF variants very likely to originate CVID: one in *LRBA* and *CTLA4* (both functionally validated), two in *NFKB1* (a large deletion and a new splice-site variant), and one in *PIK3R1* (a known splice-site variant causing disease). Thus, a minimum of 15% of the 33 cases included in this study (the 36 patients include three pairs of relatives) would have a monogenic origin of CVID. Among the LoF variants described in proteins interacting with CVID candidate genes (Table 2), a new LoF variant in *MAPK8* is also a good candidate variant. *MAPK8* shows high essentiality and haploinsufficiency prediction scores and is thought to play a key role in T cell proliferation, apoptosis, and differentiation (65–67). This stop gain variant is not found in genetic databases although it affects a base with a very low GERP value. We have also described the presence of LoF variants in the genes *CR1, IBTK*, and *NCOR2* (Table 2) that have been related to B cell development and activation (68), agammaglobulinemia (69), and lymphoma (70, 71), respectively. However, *CR1* and *IBTK* show low predicted haploinsufficiency values and the cases described at *NCOR2* follow a recessive model for the disease.

In addition to these LoF mutations at candidate CVID genes and interacting proteins, we propose other possible monogenic cases produced by missense variants at CVID candidate genes following a dominant (*PRKCD, CLEC16A, DOCK8*) or recessive models (*CR2, PLCG2*), as well as in other genes not previously associated with CVID (*KMT2C*). Of interest, missense variants and deletions in *PLCG2* with dominant inheritance have been related to PID in previous studies (72, 73). RVIS scores are also negative in these three genes which suggests a certain level of intolerance to mutations, although in the case of immunological diseases this value seems to be less indicative than for other diseases (32). Finally, two affected sisters (N205 and N206) harbor a new missense variant in *PIK3R1*. This variant has not been previously reported and is located in a conserved nucleotide according to its GERP value (3.24), although it is not predicted to be damaging using SIFT and Polyphen. On the situations fitting a recessive model, for the *PLCG2* gene, one of the variants is predicted to be damaging with Polyphen and also shows a very high level of evolutionary conservation, although for the second variant both the evolutionary conservation and predicted phenotypic effect are low. Similarly, only one of the variants at *CR2* in patient N212 shows a high level of evolutionary conservation, and none of the two variants is predicted to be damaging with Polyphen, being therefore a less promising candidate to originate CVID. Finally, *KMT2C* encodes for a nuclear methyltransferase (MLL3) of the mixed-lineage leukemia family the genes of which are among the more frequently mutated in cancer (74); somatic mutations at MLL3 have been related to different types of cancer (75), while in activated B-cells, deficiencies in the MLL3–MLL4 complex have been shown to manifest defective immunoglobulin class switching (76).

Thus, the proportion of CVID monogenic cases described in this work would rank from 15 to 24% or higher (Figure 4), similar to what has been described in previous studies (12–14) although lower than the 40% proposed in a recent analysis of 278 PID families including 20 CVID cases (77) (Table 10). However, these studies follow differing filtering strategies and stringency criteria making the results to be only roughly comparable between them. Overall, the fraction of monogenic CVID cases seems to be slightly lower to that described in other PID (78, 79), with some recent analyses showing considerably higher detection rates of PID monogenic cases (77, 80) which is especially high in a study of severe combined immunodeficiency (SCID) (81) (Table 10). The higher percentage of Mendelian patients described in some other PID (77, 80) and especially SCID (81) is probably because of a higher severity which is also expected to correlate with the number of Mendelian cases (15). However, it is important to highlight that different factors can contribute to an underestimation of the Mendelian cases in CVID in comparison to other PID. First, because of the clinical heterogeneity of CVID it is not recommended to apply the standard exome sequencing strategy where candidate genes are compared across patients to identify as causal the gene present in several patients (82). Because of that, we have used a conservative approach by mainly considering a list of candidate genes, and used genetic variants characteristics (evolutionary conservation, Polyphen values) and gene features (haploinsufficiency, essentiality or tolerance to functional variation) mainly to indicate but not conclusively exclude a given candidate gene. For example, filtering by genic intolerance to functional variation is more effective in detecting false-positive rather than identifying the causal gene since it is known that genes producing Mendelian diseases show from medium to high intolerance values (83). Second, because of the higher prevalence of CVID compared to other PID, the use of too stringent frequency filters is not recommended, which hinders the identification of causal genes by increasing the number of candidates. And third, exome and even genome sequencing have some limitations that may produce false negatives because of the difficulties to detect structural variation. However, based on our results, the contribution of CNVs to monogenic CVID cases would be quite limited, in contrast to a more important role for common CNVs proposed in previous studies (84, 85). We have used one of the highest density array optimized for CNV detection (86), and detected only a candidate CNV consisting of one big deletion including, among others, the *NFKB1* gene. Similarly, in the recent whole-exome sequencing analysis of 278 PID families CNV represented 8% of the likely causing mutations, but no causal CNV was found among the 20 CVID patients (77).

**Table 10:** NGS studies on common variable immunodeficiency (CVID) and other primary immunodeficiency (PID).

Study	Syndrome	Approach (Coverage)	*N*	Mendelian cases^a^	Functional study
Maffucci et al. (13)	CVID	WES (NA), 269 genes(NA)	50	15	No
van Schouwenburg et al. (12)	CVID	WGS (27-40X)	34	NA	RNAseq
This work (2018)	CVID	WES (120X), CNV	36	5–8	*CTLA4, LRBA*
Gallo et al. (79)	PID	571 genes (580X), WES (>10X)	45	27, 18	10 Genes
Stoddard et al. (78)	PID	173 genes (305X)	120	18	No
Stray-Pedersen et al. (77)	PID, CVID	WES (>100X)	278, 20	110, 8	No
Al-Mouse et al. (80)	PID	162 genes (461X)	139	35	No
Yu et al. (81)	SCID	196 genes (1000X)	20	14	No

*^a^Reported in the original study*.

*NA, not available*.

Independently of the exact proportion of monogenic cases in CVID, in an important percentage of patients the disorder remains genetically uncharacterized, and it seems clear than other possible models beyond the monogenic scenario should be considered. A genome-wide association study performed on 363 CVID patients has revealed susceptibility factors in MHC and ADAM, among others (84), but association with common variation seems to be far from explaining all non-monogenic situations. As has been proposed for complex disease, this CVID *missing heritability* (87) must be hidden under other models that have not been deeply explored, as oligogenic, accumulation of rare functional variation, epigenetic (11, 88) or even somatic (89). In fact, the prevalence of CVID would fit with a model where the disease is produced by mutations in two or in a few genes, an intermediate scenario between the very rare disorders originated by a single locus and common disease produced by the interaction of many genes and environmental factors (90). Other features, such as different penetrancies and severities or the phenotypic variation in affected families, could also suggest an oligogenic origin for CVID, where the disease is caused or modulated by a few genes (91). Thus, we have performed different approaches to explore the possibility of CVID cases being originated by genetic variants in two or several genes.

Considering the digenic model, we have combined exome sequencing with PPI data, and described cases of patients with rare functional variants in CVID candidate genes and an interacting protein. Although promising, to date the number of reported examples in the literature with pieces of evidence of digenic inheritance remains quite low (62), probably because of difficulties in statistical and mainly functional analyses to demonstrate a real role in the disease (63). We have used a prudent approach based on the existence of physical interactions between proteins, to produce a reduced number of candidate interactions. Other tools to identify related genes, as the human genome connectome (92, 93) or GIANT (94) could also be used. However, since interactions predicted by these tools are based both in physical and functional associations, the number of candidate protein pairs would be higher. Still at the individual level, we have considered a polygenic model and hypothesized that CVID in a particular patient might be produced by an accumulation of rare functional genetic variants in genes related to the same function, producing a list of patients with an excess of genes with functional variants in the same immunological pathway. Finally, we have performed tests of association of rare genetic variants to disease. In this case, the goal is not proposing candidate gene(s) in a particular patient but to detect genes enriched for rare functional variation in the cohort of CVID cases compared to healthy controls. Interestingly, most of the genes with significant results in these analyses (Figure 4) are among the ones with more pieces of evidence of being related to primary immunodeficiencies (51), thus supporting their role in the etiology of CVID. However, the application of these cohort approaches can be limited to syndromes as CVID because of its genetic heterogeneity. Instead, the use of higher levels of association such as pathways or functionally related genes can reduce the genetic heterogeneity and increase the detection power.

The detection of somatic genetic variants from exome sequencing data is not straightforward. The detection power ultimately depends on the mutation frequency in the tissue, which is conditioned by the cell populations affected by the mutation and their relative abundance in blood, and will be, therefore, practically undetectable if present in low-frequency cell populations. On the other hand, high-frequency mutations present in more than 40% of the reads cannot be differentiated from germline mutations unless very high coverages are achieved. In addition to high coverages, the modification of standard NGS data analysis pipelines, which by default discard genetic variants in allelic imbalance, is required. We have tentatively analyzed exome sequencing data generated in this study (with 120X is the higher for CVID produced to date) scanning for low frequency variants with predicted high impact in our set of candidate genes. Not one of the patients presented a candidate somatic variant in any of the 97 CVID genes. A previous study proposed no role for somatic CNV in CVID, based on the stability of the overall CNV burden over time (85). However, for a proper analysis of the role of somatic variation much higher sequencing coverage would be needed, and the possibility of sequencing different cell populations or tissues with different origin could be also considered since variant callers for somatic variant calling are optimized for the comparison between healthy and affected tissue (tumor). We also propose that, as a change to the experimental design of our study, late onset CVID cases should be included in a study targeting somatic variation. Finally, epigenetics is also suspected to contribute to CVID. Altered epigenetic profiles are known to be related both to common and rare genetic disease (95). However, although epigenetics is known to play an important role in B lymphocyte differentiation and activation, there is less evidence of their involvement in PID (96). Interestingly, it has been proposed that the hypermethylation of important B lymphocyte genes has a role in CVID, through the analysis of monozygotic discordant twins (88). Thus, methylation could explain some of the many cases of CVID with intermediate penetrances, and also suggests an important role of mutations affecting gene expression (mostly not detected in exome sequencing approaches) in CVID.

We think that CVID is a main example of rare disease where it is possible to arrive at similar phenotypes by several different genetic defects, either by mutations in different genes or by different genetic mechanisms including from monogenic to epigenetic scenarios. After the success of new sequencing technologies, and in particular of whole-exome sequencing in unraveling the molecular mechanisms of many rare syndromes, rare diseases such as CVID that do not completely fit with a Mendelian model represent a new challenge for medical genomics. In this manuscript, we have proposed different approaches to the analysis of CVID from whole-exome sequencing data, and have shown its power and limitations as a diagnostic tool for the study of these diseases. Beyond the identification of the causal gene in some patients, we hope that these kinds of studies can also be used to help detect key pathways related to the development of the disease, thus contributing to a better understanding of its etiology. From our and previous results, we conclude that in an important proportion of patients it will be essential to integrate data from different omic approaches to solve the genetic origin of the disease.

## Ethics Statement

This study was carried out in accordance with the recommendations of the “Guidelines on the Informed Consent” of the Bioethics Committee of Catalonia (Departament de Salut, Generalitat de Catalunya) with written informed consent from all subjects. All subjects gave written informed consent in accordance with the Declaration of Helsinki. The protocol was approved by the Comitè ètic d’Investigació Clínica-Parc de salut Mar (Barcelona).

## Author Contributions

FC and MJ conceived the project. LA, MJ, and FC designed the study. GV-I coordinated the bioinformatic analysis. GV-I, AE-S, and EAG-N performed the functional validation experiments. All the authors participated in the analysis of the data. GV-I, AE-S, MP, PS-P, RC, LA, MJ, and FC wrote the manuscript.

## Conflict of Interest Statement

The authors declare that the research was conducted in the absence of any commercial or financial relationships that could be construed as a potential conflict of interest.

## Footnotes

^1^https://www.omim.org/ (Accessed: September, 2015).

^2^http://www.picard.sourceforge.net (Accessed: January, 2016).

^3^http://evs.gs.washington.edu/EVS/ (Accessed: April, 2016).

^4^https://omim.org/ (Accessed: September, 2015).

^5^http://omim.org (Accessed: September, 2015).
