# Supplementary material for: Evaluating the Genetics of Common Variable Immunodeficiency: Monogenetic Model and Beyond
Source: Front Immunol. 2018 May 14;9:636. doi: 10.3389/fimmu.2018.00636 (PMC5960686; doi:10.3389/fimmu.2018.00636)

Table S1. KEGG pathways considered in the study.

**Pathways**	**Genes**
B cell signaling (BCSP)	72
Cytokine-cytokine receptor interaction (CCRI)	295
JAK-STAT signaling (JSSP)	157
NFKB signaling (NFKBSP)	122
PI3K-AKT signaling (PASP)	292
Primary immunodeficiency (PIDP)	49
T cell signaling (TCSP)	103
Mismatch repair (MRP)	31
mTOR signaling (mTORSP)	159
Tumor necrosis factor signaling (TNFSP)	110
Hematopoietic cell lineage (HCL)	95
Antigen processing and presentation (APP)	78
Complement and coagulation cascades (CCC)	79
Cytosolic DNA sensing (CDNASP)	64
Chemokine Signaling (ChSP)	187
Fc epsilon RI signaling (FCERISP)	68
FC gamma RI-mediated phagocytosis (FCGRIMP)	93
Intestinal immune network for IgA production (IINIGA)	50
Leukocyte transendothelial migration (LTM)	118
Natural killer cell mediated cytotoxicity (NKCMC)	135
NOD-like receptor signaling (NLRSP)	56
Platelet activation (PAP)	122
RIG-I like receptor signaling (RILRSP)	70
Ras signaling (RSP)	229
Toll like receptor signaling (TLRSP)	106

Table S2. Number of genetic variants in the individuals included in the study.

id	NS het	NS hom	Inframe het	Inframe hom	SG het	SG hom	SPL het	SPL hom	FS het	FS hom	SLStartG het	SLStartG hom	LOH	GAINS	LOSS
L283	6195	3877	236	136	78	20	138	131	122	125	34	13	12	6	7
L287	6002	3709	215	133	77	25	124	134	107	129	33	20	2	2	3
L288	6173	3740	223	139	66	24	134	123	109	121	31	22	0	10	4
L289	5995	3788	228	135	83	28	139	122	123	124	33	15	3	2	5
L290	5994	3825	216	138	73	30	128	128	116	113	29	22	no cDNA	no cDNA	no cDNA
L291	6150	3676	238	131	63	26	136	121	136	120	29	18	1	7	3
L292	6086	3794	205	138	68	18	125	134	112	122	29	21	0	10	3
L293	6152	3803	218	125	85	25	143	124	123	126	28	26	2	2	3
L294	6098	3727	215	139	78	18	132	125	117	119	29	19	13	7	5
L295	5882	4011	230	132	69	27	112	130	110	123	24	22	2	4	2
L296	6033	3801	207	131	57	31	120	128	108	127	40	19	2	1	2
L297	6071	3813	212	137	77	22	113	142	110	115	30	19	3	5	3
L298	6191	3832	224	149	69	25	113	136	108	115	28	21	2	5	2
L299	6087	3804	218	139	71	24	132	131	114	119	28	20	2	3	2
N201	6049	3827	228	141	76	24	122	136	120	114	26	20	2	4	1
N202	6138	3705	199	143	70	22	137	127	113	119	31	18	3	7	2
N203	6409	3730	215	127	76	25	139	130	113	119	31	20	2	3	3
N204	5924	3865	210	140	62	24	142	129	116	119	33	19	1	7	3
N205	6171	3786	214	134	69	22	132	131	116	123	29	20	0	10	2
N206	6139	3793	234	128	76	21	121	141	125	113	24	21	2	3	2
N207	6065	3806	221	150	79	25	131	130	123	121	32	19	2	4	3
N208	5999	3855	227	141	71	27	142	128	123	118	28	23	1	5	6
N209	5975	3896	233	130	70	23	128	122	110	127	36	22	2	4	5
N210	6159	3810	214	126	84	26	137	124	123	130	29	26	2	2	3
N211	5951	3836	219	124	66	22	129	140	117	117	28	23	2	1	2
N212	5977	3920	211	133	70	23	135	121	126	117	35	18	1	5	2
N213	5915	3805	219	146	74	25	122	134	124	116	31	18	2	2	2
N214	6053	3790	213	137	82	24	126	128	121	123	31	21	3	10	3

N215	7376	3891	226	147	72	20	136	144	145	120	47	18	18	10	4
N216	6644	4208	215	158	72	23	126	153	119	126	38	22	4	10	8
N223	6740	3987	217	126	84	26	151	111	108	99	42	18	2	5	3
N224	6443	4174	227	115	75	29	125	129	102	98	31	20	2	7	3
N225	6526	4188	221	123	68	23	138	124	104	96	35	18	0	2	0
N226	6742	4087	196	121	78	26	144	114	119	101	32	26	4	2	2
N227	6670	4128	200	120	80	18	124	129	142	85	31	24	2	4	2
N228	6625	4186	219	121	74	24	125	124	127	91	31	25	2	6	1
N229	6681	4148	199	122	71	30	121	125	106	102	28	24	2	5	3
N230	6538	4199	209	129	63	27	137	111	109	93	29	19	3	1	7
N231	6843	4067	198	117	84	24	131	122	128	107	31	18	2	3	5
N232	6604	4110	192	119	81	26	137	114	119	101	44	20	2	1	5
N233	6719	4049	202	110	62	31	148	121	102	99	41	23	0	3	3
N234	6680	4154	203	127	79	29	128	122	99	101	36	25	2	3	3
N235	6589	4119	210	128	72	23	130	122	105	95	38	17	3	1	3
N237	6632	4234	200	118	72	32	139	124	111	99	29	22	0	5	3
N246	6711	4095	205	128	89	19	118	123	108	96	36	20			

Table S3. Nucleotide variants and CNVs previously described in OMIM CVID genes.

Gene	cDNA	AA change	Genotype	Reference	Genomic position (hg19)	OMIM variant	Controls with the variant
CD19	c.972insA	.	1/1	van Zelm et al., 2006	16:28945497-28945497	107265.001	.
CD19	c.1384delGA	.	1/1	van Zelm et al., 2006	16:28948957-28948958	107265.002	.
CD19	IVS5-1G>T	.	2*0/1	Kanegane et al., 2007	16:28947472-28947472	107265.003	.
CD19	c.156G>C	p.W52C	1/1	van Zelm et al., 2011	16:28943734-28943734	107265.004	.
CD19	c.1464delC	p.P488PfsX15	1/1	Vince et al., 2011	16:28949123-28949124	107265.005	.
CD19	c.1653_1671+9del28bpins23bp	p.G551GfsX25	1/1	Vince et al., 2011	16:28950263-28950290	107265.006	.
CR2	c.1225+1G>C	.	2*0/1	Thiel et al., 2012	1:207643448-207643448	120650.002	.
CR2	c.2297G>A	p.W766X	2*0/1	Thiel et al., 2012	1:207647642-207647642	120650.003	.
CD81	c.561+1G>A	p.E188MfsX13	1/1	van Zelm et al., 2010	11:2417201-2417201	186845.001	.
TNFRSF13B	c.310T>C	p.C104R	*/1	Castigli et al., 2005	17:16852187-16852187	604907.001	yes
TNFRSF13B	c.204insA	p.L69fsX11	2*0/1	Castigli et al., 2005	17:16852292-16852292	604907.004	.
TNFRSF13B	c.542C>A	p.A181E	*/1	Castigli et al., 2005	17:16843729-16843729	604907.002	.
TNFRSF13B	c.602G>A	p.R202H	0/1	Castigli et al., 2005	17:16843665-16843665	604907.003	.
TNFRSF13B	c.431C>A	p.S144X	1/1	Salzer et al., 2005	17:16852066-16852066	604907.006	.
TNFRSF13B	c.581_582delCCinsAA	p.S194X	0/1	Salzer et al., 2005	17:16843689-16843690	604907.005	.
TNFRSF13B	c.277_231del	p.G76fsX3	0/1	Pan-Hammarstron et al., 2007	~17:16852230-16852270	.	.
TNFRSF13B	c.512T>G	p.L171R	0/1	Pan-Hammarstron et al., 2007	17:16843759-16843759	.	.
TNFRSF13B	c.121delG	p.D41IfsX43	0/1	Salzer et al., 2008	17:16855837-16855838	.	.
TNFRSF13B	c.118T>C	p.W40R	0/1	Salzer et al., 2008	17:16855841-16855841	.	1
TNFRSF13B	c.121G>C	p.D41H	0/1	Salzer et al., 2008	17:16855838-16855838	.	.
TNFRSF13B	c.236A>G	p.Y79C	2*0/1	Salzer et al., 2008	17:16852261-16852261	.	.
TNFRSF13B	c.260T>A	p.I87N	2*0/1	Salzer et al., 2008	17:16852237-16852237	.	.
TNFRSF13B	c.298insT	p.C100LfsX12	0/1	Salzer et al., 2008	17:16852198-16852198	.	.
TNFRSF13B	c.311T>G	p.C104Y	2*0/1	Salzer et al., 2008	17:16852186-16852186	.	.
TNFRSF13B	c.445G>A	p.A149T	0/1	Salzer et al., 2008	17:16852052-16852052	.	.
TNFRSF13B	c.455G>A	p.G152E	2*0/1	Salzer et al., 2008	17:16843816-16843816	.	.
TNFRSF13B	c.492C>A	p.Y164X	2*0/1	Salzer et al., 2008	17:16843779-16843779	.	.
TNFRSF13B	c.571insG	p.D191GfsX46	2*0/1	Salzer et al., 2008	17:16843700-16843700	.	.

TNFRSF13B	c.579C>A	p.C193X	0/1	Salzer et al., 2008	17:16843692-16843692	.	.
TNFRSF13B	c.736G>T	p.V246F	0/1	Salzer et al., 2008	17:16843007-16843007	.	.
TNFRSF13B	c.171G>C	p.Q57H	0/1	Almejun et al., 2012	17:16855788-16855788	.	.
TNFRSF13B	c.693G>C	p.S231R	0/1	Almejun et al., 2012	17:16843578-16843578	.	.
TNFRSF13B	c.515G>A	p.C172Y	0/1	Zhang et al., 2007	17:16843756-16843756	.	.
TNFRSF13B	c.566A>T	p.K188M	0/1	Fried et al., 2012	17:16843705-16843705	.	.
TNFRSF13B	c.61+1G>T	.	1/1	Mohammadi et al., 2009	17:16875328-16875328	.	.
TNFRSF13B	c.124C>A	p.P42T	0/1	Mohammadi et al., 2009	17:16855834-16855834	.	.
TNFRSF13B	c.659T>C	p.V220A	0/1	Pan-Hammarstron et al., 2007	17:16843084-16843084	.	1
TNFRSF13B	c.752C>T	p.P251L	0/1	Pan-Hammarstron et al., 2007	17:16842991-16842991	.	13
TNFRSF13C	.	p.del65_73_AGAGEAAL65V	1/1	Warnatz et al., 2009	22:42322256-42322279	606269.001	.
TNFRSF13C	c.62G>C	p.P21R	2*0/1	Losi et al., 2005	22:42322714-42322715	.	yes
TNFRSF13C	c.475C>T	p.H159Y	2*0/1	Losi et al., 2005	22:42321451-42321451	.	.
LRBA	c.7970T>G	p.I2657S	1/1	López-Herrera et al., 2012	4:151223857-151223857	606453.001	.
LRBA	c.5047C>T	p.R1683X	1/1	López-Herrera et al., 2012	4:151749456-151749456	606453.002	.
LRBA	c.175G>T	p.E59X	1/1	López-Herrera et al., 2012	4:151935620-151935620	606453.003	.
LRBA	c.6657_6658del	p.E2219NfsX3	1/1	Alangari et al., 2012	4:151392817-151392819	606453.005	.
LRBA	c.865_866del	p.C289CfsX292	1/1	Charbonnier et al., 2014	4:151837580-151837582	.	.
LRBA	c.2032C>T	p.Q678X	1/1	Charbonnier et al., 2014	4:151817581-151817581	606453.006	.
LRBA	c.6657_6658del	p.E2219DfsX2221	1/1	Charbonnier et al., 2014	4:151392817-151392819	.	.
LRBA	c.4334G>A	p.R1445Q	1/1	Lo et al., 2015	4:151769975-151769975	.	.
LRBA	c.522_523insT	p.K175X	2*0/1	Lo et al., 2015	4:151849694-151849694	.	.
LRBA	c.4759_4762delACTA	p.Thr1587ArgfsTer28	2*0/1	Lo et al., 2015	4:151749741-151749744	.	.
LRBA	c.4333C>T	p.R1445X	1/1	Lo et al., 2015	4:151769976-151769976	.	.
LRBA	c.3698C>T	p.S1233F	2*0/1	Lo et al., 2015	4:151773164-151773164	.	.
LRBA	c.7516C>T	p.Q2506X	2*0/1	Lo et al., 2015	4:151242490-151242490	.	.
LRBA	c.7604C>T	p.A2535V	2*0/1	Lo et al., 2015	4:151242402-151242402	.	.
LRBA	c.5501T>C	p.L1834P	2*0/1	Lo et al., 2015	4:151727440-151727440	.	.
LRBA	c.6745G>T	p.V2249F	2*0/1	Lo et al., 2015	4:151388893-151388893	.	.
LRBA	c.1963C>T	p.R655X	0/1	Lo et al., 2015	4:151818932-151818932	.	.
LRBA	c.743A>G	p.D248G	2*0/1	Lo et al., 2015	4:151837793-151837793	.	.
LRBA	c.8502-1G>C	.	2*0/1	Lo et al., 2015	4:151186965-151186965	.	.

LRBA	c.3985_3986del	p.D1329fs	1/1	Lo et al., 2015	4:151771893-151771895	.	.
LRBA	c.8137_8138insCATG	.	1/1	Rivel-Vilk et al., 2015	4:151207099-151207099	.	.
LRBA	c.7162delA	p.T2388PfsX7	1/1	Seidel et al., 2015	4:151336654-151336655	.	.
LRBA	c.8470A>C	p.I2824T	0/1	Serwas et al., 2015	4:151199036-151199036	.	.
LRBA	c.8471T>C	p.I2824L	0/1	Serwas et al., 2015	4:151199035-151199035	.	.
LRBA	c.6661T>C	p.S2221L	0/1	van Schouwenburg et al., 2015	4:151392815-151392815	.	.
LRBA	c.6628T>C	p.S2210P	0/1	van Schouwenburg et al., 2015	4:151392808-151392808	.	.
PRKCD	c.1362+1G>A	.	1/1	Salzer et al., 2013	3:53221366-53221366	.	.
NFKB2	c.2594A>G	p.D865G	0/1	Lee et al., 2014	10:104162024-104162024	164012.004	.
NFKB2	c.2564delA	p.K855SfsX7	0/1	Chen et al., 2013	10:104161901-104161902	164012.001	.
NFKB2	c.2557C>T	p.R853X	0/1	Chen et al., 2013	10:104161895-104161895	164012.002	.
NFKB2	c.2593_2600delGACAGTGC	p.A865VfsX17		Liu et al., 2014	10:104162022-104162030	164012.003	.
NFKB2	c.2600C>T	p.A867V	0/1	Brue et al., 2014	10:104162030-104162030	164012.005	.
NFKB2	c.2556_2563delCCGAGACA	p.R853AfsX29	0/1	Brue et al., 2014	10:104161893-104161901	164012.006	.
NFKB1	c.730+4A>G	p.D191_K244delinsE	2*0/1	Fliegauf et al., 2015	4:103500200-103500200	164011.001	.
NFKB1	c.835+2T>G	.	0/1	Fliegauf et al., 2015	4:103501798-103501798	164011.002	.
NFKB1	c.465dupA	p.A156SfsX12	0/1	Fliegauf et al., 2015	4:103498090-103498090	164011.003	.
IL21	c.147T>C	p.L49P	1/1	Salzer et al., 2014	4:123542021-123542021	605384.001	.
IKZF1	c.629A>G	p.C210Y	0/1	Goldman et al., 2012	7:50459466-50459466	603023.001	.
IKZF1	c.485G>T	p.R162L	0/1	Kuehn et al., 2016	7:50450301-50450301	603023.002	.
IKZF1	c.485G>A	p.R162Q	0/1	Kuehn et al., 2016	7:50450301-50450301	603023.003	.
IKZF1	c.500A>G	p.H167R	0/1	Kuehn et al., 2016	7:50450316-50450316	603023.004	.
IKZF1	c.551G>A	p.R184Q	0/1	Kuehn et al., 2016	7:50450367-50450367	603023.005	.
MS4A1	c.IVS5DS	.	1/1	Kuijpers et al., 2010	11:60231821-60231822	112210.001	.
ICOS	c.285delT	p.Leu96fsX26	1/1	Takahashi et al., 2009	2:204820584-204820585	.	.
ICOS	c.321_330del	p.F108YfsX118	1/1	Robertson et al., 2015	2:204820620-204820630	.	.
PLCG2	c.2120C>A	p.S707T	0/1	Zhou et al., 2012	16:81953154-81953154	600220.004	.
CTLA4	c.151C>T	p.R51X	0/1	Kuehn et al., 2014	2:204735350-204735350	123890.003	.
CTLA4	c.75delT	p.L28FfsX44	0/1	Kuehn et al., 2014	2:204732740-203732740	123890.004	.
CTLA4	c.567+5G>C	.	0/1	Kuehn et al., 2014	2:204736215-204736215	123890.005	.
CTLA4	c.105C>A	C35X	0/1	Schubert et al., 2014	2:204732770-204732770	123890.006	.
CTLA4	c.110+1G>T	.	0/1	Schubert et al., 2014	2:204732776-204732776	123890.007	.

CTLA4	c.208C>T	p.R70W	0/1	Schubert et al., 2014	2:204735407-204735407	123890.008	.
MSH5	.	p.Q292H	.	Sekine et al., 2007	6:31721141-31721141	.	.
MSH5	c.253C>T	p.L85F	2*0/1	Sekine et al., 2007	6:31709045-31709045	.	38
MSH5	.	p.P786S	2*0/1	Sekine et al., 2007	6:31729925-31729925	.	28
**CNVs**							
**Gene**	**Localitzation**	**Size**	**CN state**	**Reference**	**OMIM**		
ICOS	~2:204801595-204822520	1,815bp	0	Grimbacher et al., 2003	604558.001		
LRBA	4:152211739-152222852	111.1Kb	0	López-Herrera et al., 2012	606453.004		
IKFZ1	7:50435843-50452713	16.8Kb	1	Kuehn et al., 2016	603023.006		
PLCG2	16:81945680-81951607	5,990bp	1	Ombrello et al., 2012	600220.0001		
PLCG2	16:81945481-81950322	4,841bp	1	Ombrello et al., 2012	600220.0003		
PLCG2	16:81951672-81959898	8,266bp	1	Ombrello et al., 2012	600220.0002		

.

Table S4. List of CVID candidate genes used in this study.

ACP5	CD79A	IL21	PRKCD
AICDA	CD79B	IL21R	RAC2
AIRE	CD81	IL2RA	RAD50
ANP32B	CD84	IL3	RAG1
BCL2	CD93	IL4	RAG2
BLK	CECR1	IL4R	SERPINA1
BLNK	CLEC16A	INFG	SH2D1A
BTK	CR2	IRF2BP2	STAT1
BTLA	CTLA4	ITCH	STAT3
CARD11	DOCK8	LRBA	TNFRSF13B
CASP8	FCER2	MBL2	TNFRSF13C
CD19	FCGR2A	MLH1	TNFRSF17
CD20	FOXP3	MS4A1	TNFRSF7
CD22	HLA-DQ	MSH2	TNFSF10
CD24	HLA-DR	MSH5	TNFSF12
CD27	ICOS	NFKB1	TNFSF13
CD274	ICOSLG	NFKB2	TNFSF13B
CD276	IGHM	NOD2	UNC93B1
CD37	IGKC	ORC4L	UNG
CD38	IGLL1	PDCD1	VAV1
CD40	IKZF1	PDCD1LG2	VDR
CD40LG	IL10	PIK3CD	XIAP
CD5	IL10RA	PIK3R1	
CD72	IL10RB	PLCG2	
CD74	IL12	PMS2	

Table S5. Genes with LoF genetic variants (M AF < 0.001) in each individual.

Sample	Genes[a]
L283	*OR52N1, LRBA, AKD1, AC009113.1, GPR161*, NLRP4, FAM166A, SPTBN5, NRN1L, JMJD8, DEFB132, MDP1, SMEK1, AP002478.1, ZDHHC19, GPSM1, KLK10, AC009113.1, NBPF3, MZT2A, GADL1, FAM9C, LIPJ, ZNF812, STXBP2, OVGP1, STXBP2, GEMIN8P4, SSH2, FCN3, PUSL1, SLC26A10, NMNAT3, AC073343.1, RP11-680F20.9, CFHR1, EPCAM, MEI1, AC009113.1, AKR7A2, RP11-297N6.4, MFF, C10orf68, TMEM8A, CASC3, PRKD3, CTA-299D3.8, ACPP, SLC15A2, DGKG, TLR1, MYOM2, FAM166B, AC018755.11, MYO1A, CCDC90B, HIF1AN, ZUFSP, NCAPD3, SPRYD3, AC009113.1
L287	*GRP, PDGFRB, AC187648.1, SKOR2, NDUFB5*, RIPK4, FAM179A, PLXNB2, DUSP27, TGFBR3, OPN4, SLC3A1, PCGF3, GRM6, C7orf64, PMPCB, PRPS1L1, C17orf65, APLF, C20orf78, CCAR1, ITIH5, GADL1, FLT4, MGAM, STXBP2, ACSF3, RP11-163O19.1, GJB4, OR52N1, RP1-239B22.1, ZNF226, PRPF3, ST8SIA3, GOSR1, PDHA1, C10orf93, RP11-161H23.5, CCNYL2, SPAG17, ZNF438, PVRL1, AC126323.1, ATP8B4, WWOX, BCS1L, PPP4R1L, HELQ, VPS13A, DDX58, TUFM, C2orf50, ZAN
L288	*RP11-108K14.4, PRB3, RP11-108K14.4*, UPF3A, CNGA1, ZNF778, RP11-348B17.1, C1orf189, KIF18A, OR8I2, OR8I2, MDP1, ZNF527, C5orf15, RABGGTB, ZNF599, MZT2A, DGKD, STXBP2, ZNF28, ZNF784, RP11-163O19.1, C19orf51, ZFHX4, FCN3, NHLRC2, OR51G1, DENND1C, OR2V2, LILRA1, PREPL, C10orf136, ST8SIA3, CT62, AC009113.1, ATXN3, AC002511.1, C11orf40, CCNYL2, PRUNE2, CAPZB, C10orf31, FAM180B, SYCE1L, ORAI3, SSC5D, MED15, PCDHA2, CMAHP
L289	*HEY1, HEY1, RABGGTB, AKD1*, DNAH12, PALM, PAPLN, YME1L1, ZNF527, ZNF846, ZSCAN1, ZDBF2, LSAMP, SLC22A14, ZNF660, VHL, POP1, MAMDC4, PCDHGA8, ITIH5, STXBP2, MTCH1, HDAC1, AVPI1, FAM71E2, PRSS55, RP11-163O19.1, SMOC2, CHPT1, ECM2, MUC20, RP1-239B22.1, IRAK3, TCL6, OSBPL7, ADAM33, KIAA1407, TMEM85, TTC8, CAPN11, EPS8L2, AC187648.1, CADPS, PSMD3, AC009113.1, RP11-360L9.4, C2CD4D, AC009113.1, CCNYL2, AC009113.1, CADPS, SPAG17, PDIA2, ODF1, OR13A1, RELT, SLC48A1, LATS2, WWOX, CRAMP1L, SRRM5, NDUFB5, XPO5, FAM166B, DEFA6, HCN3, OR6C76
L290	*ITIH5, FLT4, AC187648.1, ITIH5L*, TAS2R31, CCDC155, TMCO4, TCTN1, MSLNL, SLC12A4, SIGLEC9, DIDO1, PMM1, DEK, AL590708.2, KLK10, CCAR1, TAMM41, MZT2A, MTCH1, C15orf42, RP11-163O19.1, MANEA, ENDOG, C1orf222, PRR25, AC073343.1, DNAH17, PRPF3, BAIAP2L2, MEI1, GOSR1, MICALCL, FBXL17, MXD4, SUN1, UBXN11, ATXN3, ODF1, SEC16B, OR52D1, CLYBL, STRCP1, PKD1, NDUFB5, WWTR1, VGF, FAM164C, OR6C76, ERGIC1, CPNE1, MSLNL
L291	*AP002478.1*, TMPRSS12, SERPINB4, ZNF101, ZNF527, ADAM33, CARD6, SLC25A2, GPSM1, PRB3, SPATA20, STXBP2, STXBP2, BAIAP2L2, AC091435.2, RBMXL3, RPS24, OR2AG2, MUC16, PPIC, AC073343.1, TMEM85, ENPP3, WDR72, ST8SIA3, CADPS, VWDE, GOSR1, CROCCP2, PCSK5, KCNIP3, ENTPD2, AC009113.1, AC009113.1, AC009113.1, C14orf118, CCNYL2, ODF1, CCNYL2, HAS1, ISG15, SEC16B, CPNE7, NDUFB5, GAK, CYP3A5, TNRC18, AC009113.1, TPK1, ETFB, TMPRSS9, SHANK2, OR6C76, FAM151A, GP6, ERGIC1, ZNF425, GPR161, AC009113.1
L292	*STXBP2, CCNYL2*, TLR1, ROBO4, UBE2H, AC126323.1, OR8I2, OR8I2, RAD9B, TMPRSS12, PROZ, FAM154B, TRPM1, EME2, DNAH2, C19orf25, ZNF527, KRTAP24-1, HEY1, HEY1, ZNF7, IFT140, EZH1, ZNF655, GRP, MXD4, SLA2, PRB3, RP11-163O19.1, CRIPAK, MS4A12, C7orf50, AC187648.1, NDUFV2, GOSR1, AC009113.1, AC009113.1, ODF1, CCNYL2, HAS1, TTC39A, OR4A16, NOD2, PICK1, COL6A5, CWC27, RPL7A, AC018755.11, OR1J2, OR6C76, ERGIC1, OR4D1
L294	*STAB1, AC006156.1, RP11-297N6.4*, DHRS9, C3orf27, GPATCH4, IDE, OR51B5, ABCA10, ABCA10, CCL16, ZNF527, GRM6, HEY1, HEY1, IFNK, IFNK, RP11-10J21.3, BEST3, DIRC3, STXBP2, STXBP2, BAIAP2L2, EPPK1, TEX15, CHPT1, GJB4, PUSL1, MED16, ACADL, AC187648.1, NDUFV2, ST8SIA3, MEI1, OR6P1, TSPAN16, ATXN3, ANKRD28, CCNYL2, PRUNE2, ODF1, PVRL1, SYT1, DNAAF2, BAHCC1, PROM2, PPP4R1L, ROS1, RCN2, SLC19A3, FAM151A, ATP2C2, AC009113.1
L295	*GPR31, ZCCHC4, AC073343.1, CAPZB, OR6C76*, USP45, AL136115.1, PTGER3, OR8I2, OR8I2, ZNF527, CCDC150, ZNF7, GPSM1, DPEP3, STXBP2, STXBP2, MC2R, PRB3, CDC27, PHF21A, GJB4, H6PD, OR51G1, PPIC, C10orf136, ST8SIA3, ITIH5L, GOSR1, PDHA1, AC009113.1, TSPAN16, ANKRD28, C11orf40, CCNYL2, AC009113.1, JPH3, RP11-297N6.4, SPAG17, OR4A16, CASC3, RPL18, APOBEC3B, ROBO2, AC009113.1, MARVELD3, TTLL6, AC009113.1
L296	*FLT4, ST8SIA3, SKOR2*, PCDHB13, AMICA1, TAS2R19, CXCL10, VPS41, WDPCP, MRPL37, OR51B5, OR8I2, OR8I2, BDNF, CNGA4, MYH1, MYH8, SPATA4, CLK4, MACC1, HEY1, HEY1, BOC, ARL9, NBPF3, PRB3, STXBP2, BAIAP2L2, SMOC2, AC091435.2, OR5B3, RP1-239B22.1, AC073343.1, EPS8L2, AC187648.1, CCNYL2, AC009113.1, MFF, ODF1, FCRL5, C10orf93, OR4A16, CD300LD, UPP2, SPTBN1, IGSF10, DSP, C6orf145, ETFB, NDUFAF1, AC007405.2, ADAM10, CMAHP
L297	*RP11-748L13.2*, ANO2, NOTUM, NFKB1, AC027045.1, OR4L1, AC022415.1, C19orf34, TXNRD3NB, HEY1, HEY1, AL590708.2, HAS1, MZT2A, TPRXL, FLT4, UBXN11, RP11-163O19.1, STXBP2, AC091435.2, OR5B3, HTRA4, MUC20, ST8SIA3, PRPF3, CADPS, AKD1, C4orf49, CROCCP2, AC009113.1, ATXN3, C14orf118, AC009113.1, PDIA2, ODF1, CCNYL2, PBXIP1, NDUFB5, ZFP2, DSP, KIAA1919, DACT2, SLC26A6, AC009113.1
L298	TAS2R19, ARGFX, RRP8, RPUSD2, AP002478.1, ENOSF1, ZNF527, NCL, AC073343.1, ABCA5,

	COL16A1, ORC3, IGSF9B, SRCRB4D, BAIAP2L2, AC091435.2, PHF21A, TTC24, RP1-239B22.1, AC073343.1, OR2V2, UNC13A, ASGR2, CTNND2, GOSR1, ATXN3, C14orf118, AC009113.1, CD300LF, MAPK8, POLN, PPAPDC3, CCDC90B, OR6C76, CMAHP
L299	*FLT4*, WDSUB1, COG7, HIST1H4E, DNAH14, OBSCN, LPAR5, OR2AP1, EME2, BEAN1, KRT34, ZNF527, ADAM33, ZNF474, SYTL3, FRMPD1, AKNA, DENND4C, RABGGTB, IQCJ, CATSPER3, MZT2A, STXBP2, MOGAT1, PCSK9, SMOC2, OR5B3, CHPT1, H6PD, CCNYL2, FGFR3, TMEM85, CADPS, GOSR1, CROCCP2, PCSK5, FAM20C, ZMYND11, RELT, KIAA0528, CAPN3, NLRP12, UPP2, CTA-299D3.8, MED15, VIPR1, PCDH18, DNAH5, RP11-43D2.2, DSP, HBS1L, KIAA1984, CDK20, MED16, CNTRL
N201	*SPATA4*, SYNM, SSPO, YME1L1, OR8I2, OR8I2, GALNT9, TTC6, SMEK1, ABCC12, AP002478.1, AC018755.11, CD33, ZNF844, ADAM33, ALPK1, MYCT1, ERCC5, KB-1269D1.8, UBXN11, MS4A12, ANKLE1, C10orf136, ST8SIA3, PRPF3, LINC00482, NDUFV2, CYP2S1, AC009113.1, AC009113.1, CADPS, JPH3, SEC16B, GPR37L1, OR52D1, OR4A16, EMP1, LCMT1, MUC16, OR1J2, ERGIC1, AC009113.1
N202	*CXorf30, TMEM85, PDHA1, CADPS*, SCRIB, ATP6V0B, FHOD1, DHX33, LOXHD1, CCDC11, KDM4B, PAM, ERGIC1, GPSM1, OBSL1, PRSS44, STXBP2, GSTO1, HOXC11, CEL, RP11-163O19.1, STXBP2, STXBP2, BAIAP2L2, RALGDS, AC006486.1, PPIC, ACOX1, EPS8L2, ASGR2, CADPS, ATXN3, AC002511.1, CCNYL2, AC009113.1, RP11-297N6.4, LRRK1, PIK3R1, ETFB, FMO6P, GPR135, DNAH7, GPR128, ERGIC1, AC009113.1, CPNE1
N203	*PRSS55, AC187648.1, CADPS*, GPR31, MYOZ3, PLAUR, RP4-801G22.3, CLCA4, DFFB, OR52I2, GALNT9, RP11-348B17.1, WWOX, PPAN, SIGLEC16, ZNF345, RDH8, KIAA1841, NCL, ZNF474, GPSM1, KRTAP1-5, CCAR1, ANKRD27, PRSS44, GADL1, RP11-271M1.2, OVGP1, STXBP2, TTC24, PUSL1, ATP1A4, ZNF418, AC073343.1, AC073343.1, OR2V2, MEI1, CROCCP2, PCSK5, ANXA13, MMP8, FAM20C, CADPS, PDIA2, ODF1, RELT, MUC19, SSPN, RPL18, CTA-299D3.8, FIS1, S100A1, CLTCL1, MAEA, CMAHP, ACO1, CPNE1
N204	*C1orf222, RP11-748L13.2*, PKD1L3, ZNF180, ZNF527, FXYD7, ZDBF2, DUSP18, ZNF474, DGKB, IFNK, IFNK, C20orf111, RP11-108K14.4, PGPEP1L, SRPK1, SUN3, CLEC12A, FAM71E2, METTL17, PHF21A, RP11-108K14.4, SLC26A11, ADAM33, EHHADH, C7orf50, C10orf136, PCDH15, ASB4, HP, BAIAP2L2, PLSCR1, AC009113.1, AC009113.1, WFS1, RP11-297N6.4, ODF1, FCRL5, ADCK3, TGFBR3, ZNF438, FAM111A, EMP1, CLYBL, METTL17, FAM179A, WWTR1, SURF1, DDIT4L, KIAA1755
N205	*UNC13A*, FAM153B, TCTN1, SNAPC1, C19orf25, ZNF527, FSIP2, NCL, SLC4A10, ADAM33, ABHD5, CORIN, SOSTDC1, ADCYAP1R1, HEY1, HEY1, ITIH5, TPRXL, S100A16, CDC27, OVGP1, STXBP2, OR5B3, SSH2, BET3L, PAQR8, RP1-239B22.1, FOXO3B, MARCH4, EPCAM, ITIH5L, CEP192, SPAG17, CAPZB, EML3, OR52D1, POU2AF1, HNF1A, CPNE7, RPL18, UPP2, ABHD16B, NEURL2, PLA2G3, NDUFB5, AC010170.1, DSP, FIS1, HNF1A, CCDC90B, IP6K3
N206	*CCDC90B*, FAM153B, OXCT1, PRAGMIN, RPA2, OR8I2, OR8I2, FAM55D, TCTN1, SMEK1, ABCA10, ABCA10, BIRC8, C19orf25, NCL, SLC4A10, PKDREJ, ABHD5, SPATA4, CORIN, SOSTDC1, ADCYAP1R1, HEY1, HEY1, ARHGAP32, LCA5L, AKD1, SKA3, ARL9, ITIH5, TPRXL, MAPRE2, S100A16, UBXN11, OVGP1, SMOC2, OR5B3, PRR25, UNC13A, EPCAM, CEP192, RCN2, CADPS, SPAG17, FOLR3, OR52D1, RELT, OR4A16, POU2AF1, KRT5, MYH11, LPO, MYO15B, FANCL, UPP2, NEURL2, PLA2G3, AC010170.1, FIS1, RPL7A, ETFB, RNFT2, IP6K3
N207	*AP002478.1, FLT4, PRICKLE3*, BPIFA2, TAS2R19, NFKB1, ZNF488, P2RX4, SMEK1, ABCA10, ABCA10, C19orf34, KIAA1841, SAMM50, TTLL1, ZNF852, ADAM18, SCN7A, MSLNL, GRP, TPRXL, MTCH1, ZBP1, METTL17, STXBP2, CRIPAK, C14orf177, LOXL2, CAPN11, NPM2, RNASEH2B, LINC00482, ST8SIA3, GADL1, AKD1, VWDE, OVGP1, DPP7, SRCRB4D, UBXN11, FAM194A, OVGP1, AC009113.1, HAS1, SEC16B, RELT, CLYBL, METTL17, CASC3, FBF1, CNDP1, PGLYRP2, CYP4F8, ANO7, PRSS50, DSP, SLC26A7, ZNF749, CMAHP, MSLNL
N208	*AP002478.1, OVGP1, AC009113.1, CLYBL*, TAS2R19, LAD1, MTA1, ALPL, ZNF488, EEF1G, P2RX4, ABCA10, ABCA10, C19orf34, ANO7, KIAA1841, ZNF852, ZNF7, HORMAD2, COL5A3, MSLNL, GRP, ZNF880, TPRXL, FLT4, MTCH1, ZBP1, OVGP1, SPATA20, RBMXL3, CRIPAK, C19orf51, C14orf177, CLEC11A, LILRA1, CAPN11, RNASEH2B, LINC00482, GADL1, VWDE, GOSR1, OVGP1, DPP7, PTPRQ, SUN1, HAS1, SEC16B, METTL17, CASC3, FBF1, FANCL, NEURL2, SMC1B, PRSS50, DSP, PPP2R3A, ELOVL7, CMAHP, MSLNL
N209*	SLCO3A1, TAS2R19, VWDE, LGALS8, OR4A15, OR8I2, OR8I2, SMEK1, TAC4, AOX1, SLC3A1, NCL, TTN, PIGP, DERL3, PRSS48, SPATA4, ZFP2, PCDHGA8, ALDH1B1, ZNF880, MZT2A, OVGP1, ANKRD33, DHDH, MUC20, GJB4, ANKLE1, BPI, SEPT9, HP, PRPF3, SRCRB4D, PLA2G4E, PRUNE2, HAS1, DAK, OR4A16, POU2AF1, RP11-65D24.2, OR6C76, ERGIC1
N210	*MANEA, AKD1*, SLCO3A1, TAS2R31, TAS2R19, VWDE, PHYHD1, OLFML1, OR4A15, TMEM59, OR8I2, OR8I2, AOX1, TTN, ESF1, DERL3, PRSS48, SPATA4, PCDHGA8, FGD4, ARL9, DDX60L, MZT2A, TPRXL, FLT4, OSMR, PDGFRB, CLEC12A, SCN7A, PTGR1, PRB3, UBXN11, ACSF3, ZSWIM7, EPPK1, BEST3, ANKRD33, DHDH, H6PD, ANKLE1, HP, EPS8L2, IWS1, SUN1, PRUNE2, JPH3, DAK, OR4A16, POU2AF1, CLYBL, CASC3, AC068473.1, ARHGEF38, DSP, AC009113.1, DDIT4L, ERGIC1
N211	*WWTR1*, TTN, TAS2R31, NIPSNAP3B, TAS2R19, FAM54B, HIST1H2BM, C1orf189, BAI2, OR51B5, TCTN1, MDP1, TTLL6, CTLA4, AC011298.2, KIAA1841, NCL, C2orf77, ADAM33, TRAIP, TRH,

	PRPS1L1, ZSCAN1, TPPP2, KB-1269D1.8, TPRXL, MTR, PRB3, PRSS44, OVGP1, RP11-163O19.1, SPATA20, CRIPAK, EPPK1, ENDOG, OR52N1, ST8SIA3, CUTC, RNASEH2B, LINC00482, ASGR2, CIZ1, AC002511.1, ANKRD28, SPAG17, ODF1, HAS1, CAPZB, OR52D1, EPSTI1, ANO7, TRIM7, NUP214, AC018755.11, DCK, NKD2, ABHD14B, GAD1
N212	*HEY1, HEY1, CADPS, AKD1, SKOR2*, RGL3, TAS2R19, CEACAM20, OTUD6B, PTPRH, SEC16B, C1orf189, DNAH14, KIAA1107, TPCN2, OR1S1, GALNT9, AC018755.11, KIAA1841, NCL, SPATA4, SAMD9, CLEC12A, TCP11, LLPH, UBXN11, OVGP1, STXBP2, C14orf177, RP1-239B22.1, UNC13A, LOXL2, CAPN11, HEATR6, EPS8L2, FKBP6, TMEM163, SUN1, PDHA1, TSPAN16, SPAG17, CPNE8, ARMC6, PBOV1, EEF1E1, AC009113.1, AC018755.11, FAM151A, GPR161
N213	ORAI3, DNAJC12, NRP1, TTLL6, GNLY, NCL, SGSM1, ZNF474, IBTK, HEY1, HEY1, GPSM1, DEM1, MSLNL, FLT4, PDGFRB, ACAD10, SPRY4, FAM71E2, ACSF3, OVGP1, PHF21A, CHPT1, SSH2, OR2V2, TMEM85, ESX1, C10orf136, GOSR1, TSPAN16, SETD4, SLC25A15P2, DPYSL3, C14orf118, AC002511.1, SLC25A15P2, AC009113.1, MFF, FCRL5, OR52D1, AMHR2, CDT1, GEMIN4, OSBPL1A, UPP2, PPP4R1L, SORBS2, GPRC6A, AC018755.11, OR1J2, RIPK3
N214	*DIRC3, PDHA1, RELT*, AMICA1, DNAH14, DUSP12, TNN, OR51B5, GPR182, AC018755.11, SHKBP1, KCTD18, TXNRD3NB, HEY1, HEY1, GPSM1, KRTAP1-5, RBM14, STXBP2, RAD21L1, TCP11, UBXN11, SPATA20, SMOC2, FAM111B, MUC20, PUSL1, ZNF586, LOXL2, RNASEH2B, UBXN11, C14orf118, C11orf40, OVGP1, AC009113.1, CADPS, JPH3, CAPZB, C10orf68, GEMIN4, UPP2, ANKRD43, CYP3A5, FAM166B, TPK1, C22orf39, EIF4G2, HIF1AN, OR6C76, PLAC9, CLEC11A, CPNE1
N215*	*PLA2G4E, PRICKLE3*, TAS2R31, TAS2R19, TTLL6, TRIM69, CPZ, BEND7, YME1L1, AP000679.2, FOLR2, OR51B5, OR51V1, RP11-113D6.6, OR6C4, TDP1, PIF1, NQO1, GALR2, MYO15B, C19orf34, MEIS1, DEFB119, PLCH1, MYCT1, GPER, ZNF7, IFNK, IFNK, OR13D1, RABGGTB, PCDHGA8, CENPK, CASP1, HERC2, TRIM22, RHOBTB3, TTYH2, RP11-108K14.4, ABCC12, TAMM41, NR2C2, MSLNL, TNFRSF6B, GADL1, CCDC17, RIN2, RP11-240B13.2, PRB3, STXBP2, SMOC2, AC091435.2, EPPK1, PHF21A, RP11-108K14.4, TGIF1, PRR25, AC073343.1, BPI, LOXL2, C1orf148, C2orf65, VWDE, MEI1, ABCA9, CYP2D7P1, PTPRQ, C14orf118, ANKRD28, TTC31, FAM194A, RP11-297N6.4, MFF, MMP8, SPI1, OR6C76, A2ML1, CMTM5, AC079354.1, CTA-299D3.8, HCLS1, LPL, OR13D1, DDIT4L, NCOR2, USP50, ASPSCR1, NUDT13, CCDC90B, OR4F6, MINPP1, NDUFV3, OR6C76, MT1CP, C2orf50, MSLNL
N216	*YME1L1, OR6C4, MYCT1, IFNK, IFNK, RHOBTB3, PRR25, MEI1, ZC3H12B, PRICKLE3*, TAS2R19, EPS8L1, TTLL6, TRIM69, CPZ, AP000679.2, OR51B5, OR51V1, MDP1, TDP1, PIF1, ABCA10, ABCA10, GALR2, ANO7, NCL, ZNF7, OR13D1, PCDHGA8, GJB7, CENPK, ITGBL1, AC011497.1, AC017028.1, CEP68, CASP1, HERC2, ERGIC1, IZUMO2, PLA2R1, TAF6, TAMM41, C8orf33, TNFRSF6B, GADL1, CCDC17, RP11-240B13.2, PRB3, THNSL2, AC073343.1, TMEM85, BPI, LOXL2, ST8SIA3, BAIAP2L2, VWDE, ARHGAP8, SUN1, AC002511.1, TTC31, FAM194A, CCNYL2, CADPS, SPAG17, MFF, CCNYL2, MMP8, OR6C76, A2ML1, CMTM5, WWOX, UPP2, HCLS1, NCOR2, USP50, KPRP, MT1CP, C2orf50, EMX1, DNAH14, OR51B5, KRTAP1-5, MSLNL, ZNF812, AC091435.2, AC073343.1, SSU72, RNFT2, TPSD1, SETBP1, OR7G3, SP100, LOXL2, ITPK1, CDH23, AC187648.1, ANKRD30B, KRTAP10-3, FAM185A, AC005220.3, TNFRSF12A, C10orf93, SLC25A15P2, EXOC3L4, TCF25, SLC25A15P2, ANKLE1, DDOST, MST1L, ZNF683, C4BPB, CCDC7, MYO7A, ISCA2, TUBD1, RPL18, NUCB1, CTA-299D3.8, NF1P6, SSUH2, SENP5, BOD1L1, RRH, LPA, UNC13B, SLC6A8, BCAP31, TPK1, DGKD, MRPL28, CCT6P1, POMZP3, TCF25, TCF25, MRPL28, PDHA1, AC073343.1, RNFT2, SLC6A8, BCAP31, TREH, KRT36, AL136115.1, DNAH14, RABGGTB, KRTAP1-5, CASP1, ADAM18, MSLNL, GJB4, SETBP1, OR7G3, SP100, PITX1, RP11-43F13.1, ITPK1, LINC00482, EMX1, KRTAP10-3, FAM185A, AC005220.3, TNFRSF12A, KMT2C, ANKLE1, MST1P2, RP11-830F9.6, AC134915.1, PDE4DIP, ZNF683, C4BPB, MYO7A, DNAH3, TUBD1, CTA-299D3.8, MAATS1, HIST1H1C, HOXA1, TPK1, CHIA, MRPL28, CCT6P1, MRPL28, PDHA1
N223	EMX1, DNAH14, OR51B5, KRTAP1-5, MSLNL, ZNF812, AC091435.2, AC073343.1, SSU72, RNFT2, TPSD1, SETBP1, OR7G3, SP100, LOXL2, ITPK1, CDH23, AC187648.1, ANKRD30B, KRTAP10-3, FAM185A, AC005220.3, TNFRSF12A, C10orf93, SLC25A15P2, EXOC3L4, TCF25, SLC25A15P2, ANKLE1, DDOST, MST1L, ZNF683, C4BPB, CCDC7, MYO7A, ISCA2, TUBD1, RPL18, NUCB1, CTA-299D3.8, NF1P6, SSUH2, SENP5, BOD1L1, RRH, LPA, UNC13B, SLC6A8, BCAP31, TPK1, DGKD, MRPL28, CCT6P1, POMZP3, TCF25, TCF25, MRPL28, PDHA1
N224	*AC073343.1, RNFT2, SLC6A8, BCAP31*, TREH, KRT36, AL136115.1, DNAH14, RABGGTB, KRTAP1-5, CASP1, ADAM18, MSLNL, GJB4, SETBP1, OR7G3, SP100, PITX1, RP11-43F13.1, ITPK1, LINC00482, EMX1, KRTAP10-3, FAM185A, AC005220.3, TNFRSF12A, KMT2C, ANKLE1, MST1P2, RP11-830F9.6, AC134915.1, PDE4DIP, ZNF683, C4BPB, MYO7A, DNAH3, TUBD1, CTA-299D3.8, MAATS1, HIST1H1C, HOXA1, TPK1, CHIA, MRPL28, CCT6P1, MRPL28, PDHA1
N225*	*RNFT2, TNFRSF12A*, DNAH14, C19orf34, KLK10, ADAM18, MSLNL, ZNF812, AC091435.2, AC073343.1, SSU72, TPSD1, OR7G3, POTEI, RP11-43F13.1, LINC00482, EMX1, KRTAP10-3, FAM149B1, TNFAIP6, SLC25A15P2, C14orf118, SLC25A15P2, MICA, AC134915.1, PDE4DIP, MST1L, ZNF683, OR5M10, MYO7A, OR6C76, ISCA2, DNAH3, RPL18, NUCB1, MED15, MAATS1, SSUH2, RRH, PCDH12, PSORS1C1, CDSN, HOXA1, UNC13B, TBX10, HLA-C, DGKD, CHIA, TCF25, PDHA1
N226*	*SETBP1, PITX1, CYP2F1, SLC6A8, BCAP31*, CHIA, SMAD9, SUGP1, BEND7, OR51B5, ABCA10, ABCA10, CD33, KIAA1841, SLC3A1, DIRC3, STXBP2, TGIF1, AC073343.1, TMEM85, DHRS4L2,

	TPSD1, TCF25, SLC4A11, CDH23, EPS8L2, ANKRD30B, OBP2A, TNFRSF12A, PRKAB1, CHN1, AC002511.1, RP11-830F9.6, AC134915.1, PDE4DIP, MST1L, CCDC7, OR52D1, OR5M10, MYO7A, GALNT9, C12orf56, RP11-131H24.4, NF1P6, WWTR1, ZNF662, RP11-529F4.1, FAM13A, KCNK16, CDSN, CYP3A5, GATS, ASMTL, DDIT4L, ZNF646, GGA2, C6orf201, POMZP3, GARS, TCF25, CMAHP, MRPL28, PDHA1
N227	*CDSN, SLC6A8, BCAP31*, CHIA, GALNT9, EME2, ABCA10, ABCA10, KIAA1841, SLC3A1, ARL9, PRSS55, DIRC3, STXBP2, DHDH, RNFT2, SETBP1, SLC4A11, PITX1, C10orf136, MST1P2, ANKRD30B, BAIAP2L2, CTNND2, PRKAB1, TNFAIP6, KMT2C, PLEKHN1, ANKRD28, C11orf40, EXOC3L4, AC134915.1, FCRL5, MST1L, LIPN, CCDC7, OR52D1, MYO7A, PLEKHG6, GALNT9, RP11-131H24.4, SPG7, ARSG, CYP2F1, PRKD3, DHRS9, NF1P6, WWTR1, PCDH12, HCG27, PSORS1C1, KCNK16, INTS1, GATS, SRRM3, FAM166B, ASMTL, KCNQ5, ZNF646, MRPL28, TCF25, MRPL28, ST8SIA3, PDHA1
N228*	*ANKRD28, PCDH12, HCG27, PSORS1C1, CDSN, SLC6A8, FAM47C, BCAP31*, ST8SIA3, GALNT9, EME2, SERPINB4, SLC3A1, ADAM33, RP11-108K14.4, ARL9, PRSS55, UBXN11, RP11-108K14.4, DHDH, C19orf51, AC073343.1, RNFT2, C10orf136, MLPH, MST1P2, CDH23, LINC00482, KIR2DL3, EMX1, BAIAP2L2, VWDE, CTNND2, PARP4P2, TNFAIP6, KMT2C, PLEKHN1, C11orf40, EXOC3L4, MICA, MST1P2, FCRL5, CSF3R, MST1L, LIPN, ACBD5, MYO7A, FOSL1, PLEKHG6, CDADC1, RP11-131H24.4, SPG7, ARSG, PRKD3, DHRS9, NDUFB5, HYAL3, RP11-723O4.6, SSUH2, SPDL1, INTS1, GSAP, SRRM3, FAM166B, AC018755.11, PPP2R3A, OR1J2, EGF, CCT6P1, PDHA1
N229	*SETBP1, HCG27, PSORS1C1*, DMGDH, APOC3, MDP1, SMEK1, EME2, KRTAP1-5, RP11-108K14.4, STXBP2, LIPJ, UBXN11, SMOC2, RP11-108K14.4, C14orf177, RP1-239B22.1, DHRS4L2, TPSD1, SP100, TTC40, ANKRD30B, MEI1, OBP2A, HLA-K, PRKAB1, C10orf93, TNFAIP6, KMT2C, CIZ1, EXOC3L4, MICA, PDE4DIP, CR1, RELT, SPI1, CLYBL, CDC16, ZNF844, CYP2F1, METTL8, NINL, PPP4R1L, PLA2G3, NF1P6, WWTR1, C3orf72, SI, IFT57, PCDH12, CDSN, ASMTL, GPR176, OR1J2, CHIA, ZNF578, CCT6P1, PLAC9, SSPO, PDHA1
N230*	*DHRS4L2, TPSD1, SETBP1, HCG27*, PTAR1, JAKMIP3, KDELC2, MDP1, SMEK1, EME2, SLC22A14, PCGF3, KRTAP1-5, GADL1, LIPJ, UBXN11, STXBP2, C19orf51, RP1-239B22.1, SP100, MLPH, TTC40, CDH23, AC187648.1, ANKRD30B, C10orf93, EXOC3L4, MICA, RP11-830F9.6, AC134915.1, PDE4DIP, OR2G6, MYO7A, CLLU1, CLYBL, RP11-131H24.4, ITGB4, ZNF844, CYP2F1, SBK2, METTL8, PLA2G3, C3orf72, SI, IFT57, SSUH2, SPP1, TXNDC15, NKD2, PBOV1, NFX1, ASMTL, PPP2R3A, PARPBP, GSC2, CHIA, ZNF578, CCT6P1, SSPO
N231	*SP100, LOXL2, OBP2A, PBOV1*, CHIA, GALNT9, AC018755.11, ZNF101, SLC22A14, OR13D1, PCDHGA8, RP11-108K14.4, ITIH5, DIRC3, SMOC2, AC091435.2, RP11-108K14.4, KIAA1407, OR2V2, RNFT2, SETBP1, PITX1, RP11-43F13.1, LCNL1, CDH23, EPS8L2, LINC00482, ANKRD30B, VWDE, TNFRSF12A, KMT2C, C14orf118, ANKRD28, C11orf40, ANKRD30A, FOLR3, OR5M10, MYO7A, OR51E1, GPRC5D, NUP107, GALNT9, CLYBL, CSNK1A1L, PSMB11, PML, PSG8, ZNF726, ITSN2, PPP4R1L, RP11-723O4.6, CHL1, BOD1L1, COL25A1, ENPEP, PCDHGA1, PCDH12, NOP16, FAM184A, RFC2, CDK5RAP2, SLC39A14, TRIOBP, EPS8L2, GPSM2, DIAPH1, FAM166A, MRPL28, CCT6P1, CMAHP, GPR161, MRPL28, PDHA1, AC009113.1
N232	*SPATA20, RELT, MYO7A, HCG27, PSORS1C1*, CPN1, CHRNB4, CES5A, KIAA1841, NCL, KRTAP1-5, AKD1, RP11-108K14.4, LIPJ, MOGAT1, RP11-108K14.4, MUC20, PUSL1, RNFT2, TPSD1, SP100, PITX1, MLPH, CDH23, EPCAM, EMX1, BAIAP2L2, PGLYRP4, AC005220.3, TNFRSF12A, PRKAB1, KMT2C, C14orf118, ANKLE1, MICA, MST1P2, AC134915.1, MST1L, FOLR3, DRD4, CLYBL, RP11-131H24.4, COCH, LACTB, CYP2F1, PRKD3, CCDC39, NEK4, SSUH2, PTPN13, PCDH12, DSP, CDSN, FAM83H, OR13C2, NIPSNAP3A, LPPR3, GCN1L1, CHIA, PLA2G4D, ADAMTS8, PDHA1, AC009113.1
N233	*PITX1, TNFRSF12A, KMT2B, ST8SIA3*, FCN2, SUPT3H, DNAH14, GALNT9, MDP1, AP002478.1, ZNF101, KIAA1841, SPATA4, ERGIC1, TTC24, ENDOG, OR7G3, RP11-43F13.1, CDH23, LINC00482, NDUFV2, FAM185A, C10orf93, EXOC3L4, ANKLE1, MICA, MST1P2, FCRL5, PDE4DIP, MST1L, ZNF683, DMBT1, OR52D1, GALNT9, CLLU1, EMP1, HSPA2, NOD2, DNAH9, CCNE1, ANKRD23, DGKD, AP001468.1, CHKB, RP11-529F4.1, SPINK8, SSUH2, HSD17B13, PCDH12, SLC36A1, TDRD6, CDSN, KRBA1, FAM166B, OR13C2, AC018755.11, MMP8, KIF24, CHIA, ZNF7, TCF25, PDHA1
N234	*GALNT9, GPSM1, TPSD1, SETBP1, SSUH2, AC018755.11*, OR2T29, CDH26, CYP4B1, QPCT, IL10RA, SMEK1, AP002478.1, GNLY, LSAMP, ZNF812, CCDC17, RP11-108K14.4, PRR25, PPIC, AC073343.1, TCF25, OR7G3, RP11-43F13.1, CAPN11, NFKB1, TTC38, CDH23, ANKRD30B, OBP2A, TNFRSF12A, TNFAIP6, TMEM88B, KMT2C, C11orf40, AC134915.1, PDE4DIP, FCAMR, MST1L, KLHDC8A, C10orf113, FOLR3, HERC1, RPL18, PSG8, ZNF599, PPP4R1L, NF1P6, NDUFB5, OTOP1, PCDH12, DSP, CDSN, FAM166B, PRKACG, ETFB, ZNF812, GPR153, DIAPH1, CBR3, CHIA, CCDC104, PDHA1
N235	*SSH2, ANKLE1, WWTR1, PSORS1C1*, CHIA, SLC36A2, AL136115.1, ABCA10, ABCA10, TTLL6, ZNF474, AL590708.2, GJB7, SUN3, TCP11, ENDOG, THNSL2, H6PD, AC073343.1, SETBP1, OR7G3, MLPH, CDH23, EMX1, PIAS1, FAM149B1, AC005220.3, RP11-360L9.4, UBXN11, TCF25, ANKRD28, C11orf40, TCF25, AC134915.1, PDE4DIP, C1orf222, SEC61A2, DENND5A, DIAPH3, MYO16, GINS2, TRPV1, CLEC10A, CTD-2368P22.1, UPP2, ATP6V1B1, CIR1, SEC23B, STAB1, AADACL2, RFTN1, SSUH2, C9, CDSN, ZAN, PLEC, OR13C2, PPFIBP2, C1orf141, CDV3, PSORS1C2, KIF21A, YIPF2, CCT6P1, TCF25, TCF25

N237*	*ZNF474, SETBP1, C4B, ANKLE1, UPP2, WWTR1, SSUH2, OR13C2*, XAF1, CHIA, AURKC, SLC36A2, ZNF8, ABCA10, ABCA10, AC018755.11, KCTD18, ADAM33, AL590708.2, ZSCAN1, SUN3, TCP11, SPATA20, SMOC2, SSH2, ENDOG, THNSL2, H6PD, AC073343.1, TCF25, OR7G3, PITX1, LINC00482, ANKRD30B, EMX1, PARP4P2, TNFRSF12A, KMT2C, TCF25, AC002511.1, C11orf40, AC134915.1, C1orf222, CHIT1, MST1L, SEC61A2, RELT, DENND5A, C12orf40, MYO16, TRPV1, CLEC10A, RPL18, CYP2F1, ATP6V1B1, CIR1, THAP7, NDUFB5, TRANK1, RFTN1, C9, PSORS1C1, ZAN, PLEC, C8orf34, ASMTL, PPFIBP2, C1orf141, PSORS1C2, TRMT2A, CHIA, YIPF2, CCT6P1, PRSS3, POMZP3, TCF25, PDHA1
N246*	*ANKRD28, CDSN, TCF25, ST8SIA3*, SLC2A8, C1orf210, AL136115.1, TTLL6, CD33, KCTD18, ZNF474, GJB7, SSH2, TGIF1, CLEC11A, OR7G3, CDH23, NDUFV2, PIAS1, AC005220.3, RP11-360L9.4, SLC25A15P2, TCF25, TCF25, SLC25A15P2, ANKLE1, MST1P2, PDE4DIP, MST1L, OR52D1, DIAPH3, GINS2, STX10, CARD8, CYP2F1, CTD-2368P22.1, SEC23B, USP16, NF1P6, C22orf23, NDUFB5, WWTR1, STAB1, AADACL2, CPA6, CDV3, NKD2, KIF21A, OAS2, CHIA, IGFN1, ZAN, GPR161, TCF25, PDHA1

[a] Genes with homozygous variants are shown in italics.

Table S6. Number of rare functional genetic variants in each individual for three gene categories.

Sample	CVID[a]	PPI-CVID[b]	ALL < 0.001[c]		Sample	CVID[a]	PPI-CVID[b]	ALL < 0.001[c]
L283	3(1)	29(1)	439(18)		N210	3(0)	25(1)	372(9)
L287	3(0)	23(1)	304(11)		N211	2(0)	25(1)	326(6)
L288	4(0)	37(0)	349(6)		N212	4(0)	28(0)	339(10)
L289	1(0)	15(0)	347(12)		N213	2(0)	23(0)	322(6)
L290	1(0)	17(0)	313(10)		N214	1(0)	16(0)	314(9)
L291	0(0)	20(0)	325(5)		N215	5(0)	42(2)	532(23)
L292	5(0)	22(0)	326(6)		N216	4(0)	38(0)	448(47)
L294	1(0)	13(0)	332(12)		N223	1(0)	36(0)	392(11)
L295	0(0)	21(0)	347(15)		N224	1(0)	29(3)	363(21)
L296	4(0)	33(1)	330(10)		N225	2(0)	28(2)	403(17)
L297	4(1)	26(2)	366(14)		N226	6(0)	48(4)	397(12)
L298	1(0)	31(1)	305(3)		N227	4(0)	41(2)	447(16)
L299	1(0)	19(1)	345(7)		N228	1(0)	32(2)	514(19)
N201	3(0)	28(0)	344(11)		N229	1(0)	35(0)	383(21)
N202	2(0)	29(2)	316(8)		N230	2(0)	39(0)	394(16)
N203	1(0)	23(0)	337(10)		N231	3(0)	25(0)	428(11)
N204	4(0)	20(0)	278(11)		N232	2(0)	22(0)	359(7)
N205	4(0)	22(0)	289(4)		N233	4(0)	32(1)	400(15)
N206	2(0)	20(0)	352(7)		N234	5(0)	40(0)	387(8)
N207	3(0)	33(1)	352(13)		N235	0(0)	21(0)	386(17)
N208	2(0)	28(1)	346(9)		N237	0(0)	32(2)	419(20)
N209	3(0)	31(1)	358(5)		N246	2(0)	19(0)	385(12)

[a] CVID candidate genes; [b] Proteins interacting with CVID genes; [c] Whole exome

Table S7. Compound heterozygotes with MAF < 0.01 and MAF < 0.001 in the whole exome for all the patients included in the study.

Sample	Genes[a]
L283	*FAM186B, MYH11, SLC25A5, SDK1, CTBP2, SLC26A1, CELSR1, STXBP2, HTT, PCLO, CASC3, ZBTB10, PABPC3, D2HGDH, LRRC43, AC009113.1, DSPP, MUC19, LINC00273, SH3D21, IGFN1, MUC16, ELOVL5, ANKRD30A*, MYC, LRIG1, NPHP4, SURF6, KRT74, CINP, EPPK1, OBSCN, KIF26A, DNAH7, SLC26A10, CHPF, FLG, LCTL, DNAH12, JMJD8, DNAH3, DNAH10, FAM55D, GPR179, NBEAL2, KIAA1797, DNAH11, TUBAL3, MUC6, ZNF816, MUC4, L1TD1, PRDM9
L287	*SULT1B1, PABPC3, OR6B3, LRRC43, MUC6, ANKRD30B, ARSD, COX6A1P2, MUC16, SHROOM4, APLF*, CBS, TRIB3, C10orf93, ZAN, UQCRC1, LAMB1, MYO10, PLXNB2, MUC5B, SH3TC1, MUC12, FAM13A, FAM155A, ZSCAN5C, MYEOV
L288	*CTBP2, PCNT, TRIO, CHD3, INPP5E, DMTF1, APBB1IP, NHLRC2, COL6A3, ADRA1D, LILRA1, ANKRD30B, LINC00273, RP11-108K14.4, IGFN1, CCNYL2*, TTLL3, OR8I2, CEP250, TTN, PLXNB1, LILRB1, PDE1C, HEPH, TEP1, PABPC3, C11orf35, DSPP, RP1L1
L289	CTBP2, TRPM7, JMJD1C, DYSF, PTPRZ1, HEY1, CADPS, HRCT1, ITIH5, AC009113.1, DSPP, MUC6, ANKRD30B, COX6A1P2, LINC00273, IGFN1, MUC16, SHROOM4, CCNYL2, FHL3, SLC25A5, FMN2, TKT, CHAT, CUL9, FREM2, EPHA8, SETX, ATP12A, CEBPZ, ZNF473, DOCK6, PDIA2, PDZD7, FSIP2, UNC5B, ADAM33, CCDC104, C9orf128
L290	*ZFP106, NAV1, MAP4, UBXN11, SEC16B, KRT3, DSPP, MUC6, MSLNL, ANKRD30B, ARSD, COX6A1P2, IGFN1, MUC16, OR52D1*, TTN, ACOX3, DNAJC13, CSMD1, NEB, COL6A3, KIF20B, PKD1L1, ABCC10, ANKS3, HVCN1, SUN1, C1orf222, PKHD1, DMKN, TCTN1
L291	*CAMSAP3, CTBP2, HNRNPA0, MSH6, CPEB2, STXBP2, TNRC18, SPATA20, RPGR, SOX1, NAALADL2, AC009113.1, CEP104, LINC00273, DMKN, MUC16, CCNYL2, WDR87*, VPS13C, TSEN54, PKD2, BAHCC1, IFIH1, SH3TC1, ARMCX4, L1TD1, CEP68
L292	*PKHD1L1, CTBP2, DCHS1, ODF1, HEY1, OR4A16, AC009113.1, DNAH2, CDHR5, COX6A1P2, GOLGA6L6, LINC00273, C1orf173, CCNYL2, SPANXN4, OR8I2*, PLEC, MLH1, NUPL2, TTN, PICK1, OBSL1, TRPM1, ESYT3, MZT2B, GRM6
L293	*LAMA5, TTN, FLT4, OBSCN, APBB1IP, C4orf17, OR4A16, LRRC43, AC009113.1, DSPP, AKD1, ARSD, LINC00273, PHGR1, RP11-766F14.2, CCNYL2, C16orf89, OR8I2*, EPPK1, RARA, NEB, RERE, FMN1, MUC5B, KDM6B, PDZD2, ARNT, PKP3, FAT2, AHCTF1, MSLN, KLLN, MUC4, MUC16, MAGEC1
L294	*PRSS16, IGF2R, TTN, PCNT, STXBP2, IFNK, FEZF2, BEST3, FAM160B1, HEY1, HSPA12A, ZNF141, ACADL, PABPC3, HRCT1, PRDM6, ADM2, COX6A1P2, LINC00273, ABCA10*, TLR5, RBM28, ATP12A, SPTA1, ZFHX4, MUC4, SLC23A1, MUC16, OR6P1, FAM186A, PRDM9
L295	*CTBP2, SELPLG, STXBP2, MAGEA3, CASC3, PABPC3, HRCT1, OR4A16, AC009113.1, MUC6, AC022098.1, LINC00273, IGFN1, NHS, OR8I2*, CYP2D7P1, TLR5, MED16, ANKRD11, PODXL2, LOXHD1, CACNA1S, ANKZF1, MPDZ, MUC5B, CLSTN3, QSOX2, GJB4, C8orf73, OR10R2, MUC16, RP1L1, OVCH1, TPRX1
L296	*C10orf93, UQCC, DMTF1, ODF1, HEY1, MEFV, OR5H14, OR4A16, DSPP, PIEZO1, RP1-239B22.1, MUC16, OR8I2*, UNKL, TBL3, PLEKHA6, F3, MYH1, SETX, PTPN23, FAT2, TCTN3, FAM47A, C1orf87, ZSCAN5A
L297	*PHLDB2, LAMA5, TTN, SELPLG, DLGAP2, MUC2, UBXN11, HEY1, KIAA1919, RPGR, HRCT1, AC009113.1, ARSD, LINC00273, MUC16, TPRXL, CCNYL2*, SLC25A5, SIRT6, ATXN1, FLNB, REXO1, HECTD3, PTPN13, OPHN1, ZXDC, FMN1, RP1L1, DCHS2, EFCAB5
L298	*PDE4DIP, PCNT, ZFHX3, DYSF, PABPC3, AC073343.1, LINC00273*, BMP1, BZRAP1, NUPL2, DNMBP, MMRN1, EPHA8, TPP1, TTC39B, FAT3, MUSK, TNS1, MUC5B, RGS6, PRKD1, LRRC56, ZC3H3, UNC13C, CPA6, C1orf222, MUC16, C10orf112
L299	*ACOT4, GPR112, CTBP2, DYSF, WISP1, ATXN7, KRT4, OBSCN, PABPC3, HRCT1, ADAMTS13, MUC6, AL445989.1, ANKRD30B*, SLC25A5, UNC13C, GPR108, MTUS1, GRIN2D, TNRC18, HMCN1, ZNF77, C16orf59, VPS13A, CASS4, PHLDB1, DNAH10
N201	*ACOT4, AC019294.1, POLR1C, HNRNPA0, TNFSF9, TCHH, UBXN11, MAMDC2, PABPC3, HRCT1, PAPLN, OR4A16, D2HGDH, AC009113.1, UMODL1, ZNF469, DSPP, MUC6, ANKRD30B, GOLGA6L6, LINC00273, MUC16, OR8I2*, SLC25A5, ZFYVE26, C10orf93, TKT, STARD9, DCAF4, NUDT16, ABCA12, CPB1, FLG2
N202	*ACOT4, TTN, ERGIC1, STXBP2, CFTR, CADPS, PABPC3, CROCC, D2HGDH, AC009113.1, ARSD, GOLGA6L6, CWF19L2*, WFS1, SLC25A5, AC005609.17, MSH3, TTC39B, PRX, UBXN11, VPS13A, DNAH7, LEPREL2, SYT15, ADAMTS4, FBN3, MAGEC1
N203	*TTN, PLEC, CUL9, TARBP1, COL4A2, ODF1, CADPS, SOX1, STAB1, PABPC3, DSPP, AC073343.1, MUC16*, SLC25A5, SEPT1, SYNJ2, NOTCH3, PDIA4, IGSF9, POLQ, OTOF, TECPR2, ASXL2, SPTA1, PDIA2, VPS13A, OR10V1, B4GALNT3, DNAH12, FCRL3, PMFBP1, NBEAL2, TDRD12
N204	*ACOT4, ZAN, HNRNPA0, DYSF, IFNK, SOX1, D2HGDH, AC009113.1, AC073082.1, COX6A1P2, LINC00273, METTL17, RP11-108K14.4, ZYG11A*, FASN, ESCO2, PLEC, WFS1, NAV2, FMN1, TGFBR3, MYO15A, HKR1, PPP6R2, ADAM33, TEP1, PCDH18, CROCC, LRRC56, ZNF469, INSC, ANKS3, FCRL5, GGN
N205	*CTBP2, HNF1A, STXBP2, KCNK1, CEP192, HEY1, SOX1, RTP2, C7orf62, DSPP, ZNF91, MUC16,*

	TPRXL, PLA2G3, FAM186A, CYP2D7P1, PDE4DIP, TTN, BRCA2, CCDC90B, OTOF, TNRC18, GCOM1, HKDC1, XIRP1, RFTN2, RP1L1, MAGEC1
N206	*ACOT4, CTBP2, STXBP2, KCNK1, UBXN11, ATAD5, CEP192, HEY1, ZNF536, RTP2, OR4A16, AC073082.1, LINC00273, PRR25, TPRXL, ABCA10, PLA2G3, OR8I2*, CYP2D7P1, PDCD11, BRCA2, CCDC90B, LENG8, MUC2, MPDZ, TNRC18, SH3TC1, C9orf68, GCOM1, XIRP1, MUC4, L1TD1, OVCH1, MAGEC1
N207	*LOXHD1, MLL3, STXBP2, PRSS3, TNRC18, CYP4F8, CNN2, RPGR, SOX1, PRDM6, MSLNL, LINC00273, METTL17, MUC16, TPRXL, ABCA10, OVGP1*, AK2, RARA, TTN, DSP, GP1BA, EPHA10, UBXN11, CEP192, PABPC3, TRPM6, L1TD1, CCDC105
N208	*LOXHD1, CHD3, MLL3, TCHH, TNRC18, LILRA1, PTPRQ, MSLNL, COL5A3, TPRXL, ABCA10, OVGP1*, BDP1, RARA, TTN, DSP, FBXL6, GP1BA, MUC2, CEP192, TRPM6, MUC16, L1TD1
N209	*SLC4A7, LAMA5, TTN, HAS1, APBB1IP, PLA2G4E, HRCT1, OR4A16, LRRC43, AL445989.1, LINC00273, OR8I2*, NOTCH2, TMEM222, EPPK1, SNAPC4, ATP12A, FMN1, PDZD2, ARNT, MSLN, HLA-DRB5, ARHGAP40, KLLN, SLX4, KIAA0922
N210	*EPPK1, TTN, FLT4, UBXN11, OBSCN, APBB1IP, CNN2, C4orf17, OR4A16, LRRC43, AKD1, LINC00273, PHGR1, RP11-766F14.2, MUC16, TPRXL, C16orf89, OR8I2*, RARA, NEB, RERE, FMN1, MUC5B, KDM6B, PDZD2, ARNT, PKP3, FAT2, AHCTF1, MSLN, MUC6, KLLN, MUC4
N211	*PDE4DIP, ZFP64, KIAA0284, AHDC1, SOX1, PABPC3, CACNB2, RTP2, MUC6, ZNF91, LINC00273, MAGEC1, TTLL6*, GLTSCR1, ZAN, PARN, LAMA5, TTN, TOX, NKD2, MUC2, EFHA1, HVCN1, TCTN1, SPEF2
N212	*ACOT4, CPEB2, RHBDL2, INPP5J, UBXN11, NLRP1, HEY1, CNN2, VPS13B, AC018755.11, MUC16*, MYBBP1A, MUC2, PTPRH, ADORA2A, PLCG2, ARHGEF4, TG, PRUNE2, LARP1B, DMKN, MFSD6L, RP1L1, ZNF804B, TAF7L
N213	*ACOT4, SLC25A15P2, HNRNPA0, SETX, GEMIN4, OBSCN, HEY1, CNN2, AC073082.1, CCNYL2*, SLC25A5, TTN, RRP9, CELSR1, GRIN2C, EPHA8, TNS1, PRDM16, ZC3H4, LRRK2, ODZ4, PTPDC1, SLCO4A1, GPR98, DNAH5, L1TD1, GPRC6A
N214	*UBXN11, HEY1, CNN2, HRCT1, DNAH10, MUC16*, SLC25A5, RPN1, HMCN1, FANCM, VPS13A, FRMD1, PMFBP1, UMODL1, MUC19, DCHS2, MUC17, ZNF534, SPATA16
N215	*GRM6, C2orf3, TTN, NACA, STXBP2, TTC31, MTERFD2, TRERF1, IFNK, NRTN, TSKS, CNN2, C2orf50, PLA2G4E, SOX1, ANKRD26, PLEKHH3, PABPC3, OR5H14, A2ML1, MSLNL, MT-CYB, PHGR1, RP11-108K14.4, OR6C76, ZNF208, OR13D1, MUC16, MAGEC1*, KDM4C, PTPN13, LRP6, RRBP1, MYLK, ATM, ATG2A, HSPA6, SOX30, CELSR3, MAP4, HPS5, CAND2, COL18A1, CCDC88B, PDE4C, PIWIL4, CSRP2BP, MYH13, TIMELESS, KCNV2, MUC5B, CBX6, SPTA1, PDZD7, ADORA2A, TTYH2, CGN, COL4A3, BAG3, PRSS50, VAT1L, GADL1, ZFHX4, CSMD2, KRTAP10-5, OR2M3, TUBAL3, DNAH5, FCRL5, LINS, PRRC2B, PRSS45, IGFN1, CCDC150, SVEP1, ATXN3L, TMPRSS7, OR6C4, SLC28A3, KIAA1549, AHNAK2, ETAA1, DMP1, VWDE
N216	*PENK, NCOA3, MXD3, TTN, IFNK, INPP5J, CCDC40, ANKRD24, LGI2, SOX1, KDELC1, OR5H14, A2ML1, ARSD, PHGR1, WDR96, ZNF208, MUC16, CCNYL2, ABCA10, ZNF254*, NUP214, KDM4C, C14orf80, APOB, IGF2R, RRBP1, ATM, DYNC2H1, HSPA6, PLEKHA7, MAP4, CAND2, COL18A1, EPS8L1, TTC31, HR, MTERFD2, F13B, FAT1, SCNN1A, KCNV2, ARHGEF17, TNRC18, C17orf56, CBX6, ADORA2A, SELP, BCAS4, KIAA0317, COL4A3, DNAH1, WWOX, RHBDD1, GBP3, GADL1, KRTAP4-9, KRTAP10-5, AGXT2L2, PLEKHG4B, TUBAL3, C11orf36, FAM47A, PRRC2B, TMEM232, GJB7, COL15A1, TMPRSS7, OR6C4, SLC28A3, SLC22A10, DCHS2, KIAA1383, GALNTL5, OR5B17
N223	*GOLGA8M, HNRNPLL, RFPL4AL1, SLC25A15P2, MRPL28, SELPLG, HPR, TCF25, ATXN7, LMTK3, OBSCN, IGSF3, HRNR, ANKLE1, NBPF10, ANKRD30B, RP11-683L23.1, PCDH11Y, ESX1*, OR10X1, TTN, CUL9, ASH1L, TAS1R1, CACNA1H, LPA, MUC5B, RANBP6, ATP10A, TTC3, GPR112, PKHD1, ZBBX, SAGE1, TFF3
N224	*HNRNPLL, KMT2C, PDE4DIP, MRPL28, SELPLG, HPR, CHTF18, TOPBP1, ATXN7, KRT7, ZNF683, OBSCN, KRT36, HRNR, FAM185A, D2HGDH, ANKLE1, NBPF10, RP11-830F9.6, PCDH11Y, TCP10L2, ESX1*, OR10X1, MAATS1, RPTN, PRUNE2, ATP10A, SCUBE1, DHTKD1, TTC3, DNAH6, TEKT5, IQCE, DNAH5, GPR112, PKHD1, SAGE1, TFF3
N225	*HNRNPLL, SLC25A15P2, EP300, DOT1L, SELPLG, CHTF18, TOPBP1, ATXN7, LMTK3, HLA-C, OBSCN, SIRPB1, NBPF10, RP11-683L23.1, MUC4, PCDH11Y, TCP10L2*, PKHD1, OR10X1, MAATS1, NBPF1, MIB2, TTN, PVRL3, RANBP6, RPTN, IGSF3, SCUBE1, OR10C1, TTC3, ZNF780B, ZNF780A, DNAH6, TEKT5, IQCE, DNAH5, GPR112, PRRC2C, TTC40, MICA, ZNF546, SAGE1, TCN1, TFF3
N226	*SLC12A4, RFPL4AL1, AHNAK, NCOR2, LRBA, DDX11, SP8, BCL2L14, TCF25, ARID1B, KRT10, ATXN7, DMBT1, MUC5B, RPGR, HRNR, NBPF10, DSPP, ANKRD30B, RP11-830F9.6, MUC4, C2orf71, PCDH11Y, ABCA10*, CTD-3193O13.9, NBR1, TTN, PLEC, NTSR1, ZIC4, SASH1, ZNF462, DIRC3, IGSF3, ZNF699, PXDN, HLA-DPB1, TIGD7, MN1, CCDC88C, LINS, L1TD1, PRDM9
N227	*KMT2C, RFPL4AL1, TCERG1, LAMA5, NOTCH4, MRPL28, INTS1, HSPG2, ARID1B, ATXN7, DMBT1, MUC5B, RPGR, HRNR, NBPF10, DSPP, ANKRD30B, RP11-1407O15.2, RP11-830F9.6, MUC4, GALNT9, ABCA10, PRDM9, OR52D1, TCP10L2*, CTD-3193O13.9, NBPF1, PCNT, SASH1, SCAF1, RERE, NEFH, PDIA2, ZNF382, TPPP2, ANKRD60, ZNF788
N228	*ABCC6, KMT2C, GOLGA8M, RFPL4AL1, FOSL1, PDE4DIP, TCERG1, AHNAK, MST1P2, CRLF1, MAZ, LAMA5, TTN, ZFHX3, HSPG2, CPEB2, HLA-DRB1, SLC22A1, UBXN11, ANK2, RNFT2, MST1, VPS13B, HRNR, CCDC57, ZNF836, NBPF10, DSPP, PRRC2B, RP11-108K14.4, RP11-1407O15.2,*

	RP11-830F9.6, MUC4, VWDE, TCP10L2, CACNA1S, FBXO2, CCDC88B, RERE, ARHGEF17, NEFH, TNRC18, PDIA2, PRICKLE4, TTC40, ANKRD60
N229	*C10orf93, KMT2C, RFPL4AL1, CDC42BPG, HLA-DRB1, NEFH, HEATR5B, NYNRIN, NBPF10, DSPP, ANKRD30B, PCDHA6, RP11-108K14.4, RP11-830F9.6, SSC5D, TTC40, MUC4, KRTAP5-10, ANKRD36, TCP10L2*, NBPF1, DIDO1, GRIN3B, RHPN1, MYBBP1A, PTPN23, SYNE1, BEGAIN, KIAA0556, ST3GAL5, HRNR, ATP8B4, LRRC14B, METTL17, MUC22, OTOGL, ZNF578, ZNF816, MUC16
N230	*MZT2A, RFPL4AL1, ZAN, HNF1A, CDC42BPG, HLA-DRB1, UBXN11, NEFH, OBSCN, HRNR, AHCTF1, ATP8B4, NYNRIN, NBPF10, ANKRD30B, RP11-830F9.6, MUC4, PCDH11Y, ANKRD36, ESX1*, C10orf93, MRPS10, TTN, RNF213, HEATR1, RHPN1, PTPN23, SYNE1, PARD3B, ACSM5, OTOGL, ZNF816, CLLU1, UGT2A3
N231	*BAI1, KMT2C, MRPL28, SON, CLCN7, HLA-DRB1, EPS8L2, TOPBP1, RPGR, IGSF3, BIN2, C5orf45, ANKRD30B, RP11-108K14.4, RP11-830F9.6, GALNT9, IGFL2, TCP10L2, AC024580.1, ESX1*, TTN, MTDH, CYP2D7P1, NBPF1, TDRD7, AP3B1, SRRM2, HEXDC, IGSF22, TRIOBP, C11orf80, HRNR, FBN3, TTC40, LRGUK
N232	*KMT2C, MINK1, ETFB, NOTCH4, XYLB, IGSF3, HRNR, NBPF10, RP11-108K14.4, RP11-830F9.6, MUC4, PCDH11Y*, PMFBP1, MKNK1, CLTCL1, PRX, ESAM, TLR7
N233	*RFPL4AL1, DGKD, PER3, REPIN1, CDH23, ATXN7, ZNF7, MMP8, DNAH9, RPGR, HRNR, FAM185A, FRG1B, NBPF10, DSPP, MYO18B, AP001468.1, GALNT9, AHNAK2*, C10orf93, TTN, NAV2, KNTC1, FYCO1, FBRS, CR2, PKP4, IQUB, CECR2, PLEKHG4, COL6A5, PRRC2A, OR4C15, CLLU1, ZNF479
N234	*IGLJ3, KMT2C, PDE4DIP, NCOR2, DIAPH1, BAI1, PHLPP1, UBXN11, OBSCN, CDSN, TNS3, IVL, PCDH12, NBPF10, DSPP, ANKRD30B, RP11-830F9.6, ZNF812, PCDH11Y, OR2F1, TCP10L2, OR2T29*, CMYA5, LAMC3, MAP3K4, SPAG1, TNRC18, PCDH1, WDR66, TRIOBP, CACHD1, C2orf54, HLA-DPB1, LRRC56, EYS, KLLN, OR5L2
N235	*RFPL4AL1, AHNAK, DDX10, EBNA1BP2, CPEB2, CDH23, TCF25, PCLO, DMBT1, CDSN, SIRPB1, RPGR, ZC3H3, ANKLE1, KRTAP4-4, RP11-1407O15.2, RP11-683L23.1, MUC4, C1orf141, PCDH11Y, ABCA10, FAM149B1*, DOT1L, PLEC, ANK3, CCDC88B, MYH7B, NUFIP2, FMN1, HPS4, ZNF493, THNSL2, TRIOBP, AFTPH, HRNR, SLC7A4, GABRE, FGD5, TTC3, ANO7, SPHKAP, SLC22A18AS
N237	*KMT2C, PDE4DIP, EBNA1BP2, C1GALT1C1, TCF25, PCLO, ARHGEF17, CHIA, SOX1, ZC3H3, NBPF10, ANKRD30B, MUC4, C1orf141, PCDH11Y, ABCA10, FAM186A*, TTN, LPP, CCDC116, BZRAP1, PLEC, ANK3, CCDC88B, MYH7B, FMN1, SLC26A9, ZNF462, ECEL1, TRIOBP, RPTN, AFTPH, HRNR, TBC1D8, HLA-DPB1, ADAMTS16, PKD1L1, TEKT5, SIGLEC1, ADAM21, PRRC2C, CCDC73, ZNF479, AHNAK2, AQP11
N246	*PROB1, RFPL4AL1, SLC25A15P2, ZAN, AHNAK, DDX10, ATAD2, CPEB2, CDH23, TCF25, DMBT1, RPGR, SOX1, FAM46A, GPR98, HLA-DRB5, NBPF10, OVCA2, RP11-1407O15.2, RP11-830F9.6, MUC4, RFPL1, PCDH11Y*, SPATA31D1, ZKSCAN7, NBPF1, EIF3A, DHX38, USP53, NUFIP2, HPS4, ZNF493, B4GALNT3, HRNR, GABRE, TTC3, ANO7, SPHKAP, OFCC1, SLC22A18AS, USH2A

[a] Genes with variants with MAF < 0.001 are shown in italics.

Table S8. Gene properties for genes with compound heterozygotes with genetic variants with Polyphen > 0.5 and GERP > 2 in the patients included in the study.

Sample	Gene	Essentiality Score	RVIS
L283	FAM186B	0.02	1.41
L283	MYH11	0.69	-3.10
L283	SLC25A5	0.54	-0.03
L283	SDK1	0.24	-4.63
L287	CBS	0.98	-0.80
L287	TRIB3	0.98	0.47
L289	FHL3	0.71	0.28
L289	SLC25A5	0.54	-0.03
L289	FMN2	0.21	0.41
L291	CAMSAP3	0.11	NA
L291	VPS13C	0.45	-0.81
L292	PKHD1L1	0.11	5.45
L292	PLEC	0.95	-6.57
L292	MLH1	0.88	0.51
L293	EPPK1	0.54	NA
L294	PRSS16	0.09	-0.38
L297	SLC25A5	0.54	-0.03
L298	BMP1	0.91	-1.96
L299	ACOT4	0.17	0.28
L299	GPR112	0.11	0.08
L299	SLC25A5	0.54	-0.03
L299	UNC13C	0.15	-1.67
N201	ACOT4	0.17	0.28
N201	SLC25A5	0.54	-0.03
N201	ZFYVE26	0.40	-0.27
N202	ACOT4	0.17	0.28
N202	WFS1	0.88	-0.10
N202	SLC25A5	0.54	-0.03
N203	SLC25A5	0.54	-0.03
N203	SEPT1	0.45	0.13
N204	ACOT4	0.17	0.28
N206	ACOT4	0.17	0.28
N209	SLC4A7	0.51	-0.97
N209	NOTCH2	0.97	-1.82
N209	TMEM222	0.95	-0.12
N209	EPPK1	0.54	NA
N210	EPPK1	0.54	NA
N211	GLTSCR1	0.62	NA
N212	ACOT4	0.17	0.28
N213	ACOT4	0.17	0.28
N213	SLC25A5	0.54	-0.03
N214	SLC25A5	0.54	-0.03
N215	GRM6	0.11	-1.32
N215	KDM4C	0.95	-0.34
N215	PTPN13	0.80	1.30
N216	PENK	0.51	0.31
N216	NUP214	1.00	-2.15
N216	KDM4C	0.95	-0.34

N223	OR10X1	0.02	1.51
N224	OR10X1	0.02	1.51
N225	PKHD1	0.09	0.38
N225	OR10X1	0.02	1.51
N226	SLC12A4	0.57	-1.92
N227	KMT2C	NA	NA
N228	ABCC6	0.16	0.63
N228	KMT2C	NA	NA
N228	CACNA1S	0.83	-1.21
N230	MZT2A	0.11	NA
N231	BAI1	0.85	NA
N231	TTN	0.95	2.17
N231	MTDH	0.54	-0.42
N232	KMT2C	NA	NA
N232	PMFBP1	0.19	-0.66
N234	CMYA5	0.54	10.43
N237	KMT2C	NA	NA
N237	TTN	0.95	2.17
N237	LPP	0.85	1.05

Table S9. Patients with rare functional variants (MAF < 0.05) and GERP >2 in a CVID candidate gene and interacting proteins.

Patient	CVID gene	Variants	Interacting protein	Variants
L287	SERPINA1	1 het	CELA1	2 het
L288	MSH2	1 het	ATR	1 het
L288	MSH2	1 het	CREBBP	1 het
L288	PRKCD	1 het	CREBBP	1 het
L288	PRKCD	1 het	PDP1	1 het
L288	PRKCD	1 het	RUNX2	1 hom
L293	DOCK8	1 het	CDC42	1 het
L299	SERPINA1	1 het	CELA1	1 hom
L299	STAT1	1 het	FGFR3	1 hom
L299	STAT1	1 het	FGFR4	1 het, 1 hom
L299	NFKB1	1 het	NCOR2	1 het
L299	SERPINA1	1 het	PRSS3	1 het
N202	PIK3R1	1 het	IRS2	1 het
N202	PIK3R1	1 het	TLR2	1 het
N204	MSH2	1 het	ATR	1 het
N205	PIK3R1	1 het	AXL	1 hom
N205	PIK3R1	1 het	TYK2	1 het
N206	PIK3R1	1 het	AXL	1 hom
N207	SERPINA1	1 het	PRSS3	2 het
N207	PIK3CD	1 het	RALY	1 het
N208	SERPINA1	1 het	PRSS3	1 het
N208	PIK3CD	1 het	RALY	1 hom
N210	DOCK8	1 het	CDC42	1 het
N213	PLCG2	1 het	FLT1	1 het
N216	PIK3CD	1 hom	RALY	1 hom
N227	NOD2	1 het	ERBB2IP	1 het
N227	PIK3CD	1 het	IRS2	1 het
N227	PIK3CD	1 het	RALY	1 het
N229	SERPINA1	1 het	CELA1	1 het
N229	PIK3CD	1 het	RALY	1 hom
N233	CR2	1 het	FHOD1	2 het
N234	RAD50	1 het	NBN	1 het
N234	NFKB1	1 het	NCOR2	2 het

Table S10. Genetic variants in genes showing an excess of rare functional variation in CVID patients compared to controls.

Gene	Sample	Genotype	chr	position	ref	alt	sift	polyphen	rs	gerp	esp5400_ea	GMAF	effect
CLEC16A	L293	0/1	chr16	11038605	G	A	1	0.012	rs202188885	4.59	.	.	MISSENSE(G11R_CLEC16A)
CLEC16A	N209	0/1	chr16	11038605	G	A	1	0.012	rs202188885	4.59	.	.	MISSENSE(G11R_CLEC16A)
CLEC16A	N210	0/1	chr16	11038605	G	A	1	0.012	rs202188885	4.59	.	.	MISSENSE(G11R_CLEC16A)
CLEC16A	L292	0/1	chr16	11073195	C	T	0.99	0.857	rs74163607	5.3	0.00	0.00	MISSENSE(R305W_CLEC16A)
CLEC16A	N227	0/1	chr16	11073195	C	T	0.99	0.857	rs74163607	5.3	0.00	0.00	MISSENSE(R305W_CLEC16A)
CLEC16A	N232	0/1	chr16	11114170	C	T	0.1	0.481	rs74163614	0.601	0.00	0.00	MISSENSE(T475M_CLEC16A)
PRKCD	N205	0/1	chr3	53215280	G	A	0.9	0.083	.	-4.35	0.00	.	MISSENSE(V125M_PRKCD)
PRKCD	N206	0/1	chr3	53215280	G	A	0.9	0.083	.	-4.35	0.00	.	MISSENSE(V125M_PRKCD)
PRKCD	L288	0/1	chr3	53218928	G	T	0.96	0.733	.	5.91	.	.	MISSENSE(V276L_PRKCD)
DOCK8	L289	0/1	chr9	289577	C	T	0.85	0.913	.	0.19	0.00	.	MISSENSE(R66W_DOCK8)
DOCK8	L293	0/1	chr9	377046	G	A	0.95	0.868	rs148693111	5.71	0.00	.	MISSENSE(V759M_DOCK8)
DOCK8	N210	0/1	chr9	377046	G	A	0.95	0.868	rs148693111	5.71	0.00	.	MISSENSE(V759M_DOCK8)
PLCG2	N212	0/1	chr16	81939089	T	C	0.93	0.598	rs187956469	5.18	0.00	0.00	MISSENSE(Y482H_PLCG2)
PLCG2	L296	0/1	chr16	81942175	A	G	0.98	0.005	rs75472618	-6.5	0.01	0.01	MISSENSE(N571S_PLCG2)
PLCG2	N203	0/1	chr16	81942175	A	G	0.98	0.005	rs75472618	-6.5	0.01	0.01	MISSENSE(N571S_PLCG2)
PLCG2	N212	0/1	chr16	81942175	A	G	0.98	0.005	rs75472618	-6.5	0.01	0.01	MISSENSE(N571S_PLCG2)
PIK3CD	N207	0/1	chr1	9777090	T	C	0.61	0.119	.	5.08	.	.	MISSENSE(M285T_PIK3CD)
PIK3CD	N208	0/1	chr1	9777090	T	C	0.61	0.119	.	5.08	.	.	MISSENSE(M285T_PIK3CD)
PIK3CD	N223	0/1	chr1	9780836	T	G	0.02	0.012	rs201274224	5.29	0.00	.	MISSENSE(S544A_PIK3CD)
PIK3CD	N224	0/1	chr1	9780836	T	G	0.02	0.012	rs201274224	5.29	0.00	.	MISSENSE(S544A_PIK3CD)
PIK3CD	N229	0/1	chr1	9780836	T	G	0.02	0.012	rs201274224	5.29	0.00	.	MISSENSE(S544A_PIK3CD)
PIK3CD	N231	0/1	chr1	9780836	T	G	0.02	0.012	rs201274224	5.29	0.00	.	MISSENSE(S544A_PIK3CD)
PIK3CD	N233	0/1	chr1	9780836	T	G	0.02	0.012	rs201274224	5.29	0.00	.	MISSENSE(S544A_PIK3CD)
PIK3CD	N234	0/1	chr1	9780836	T	G	0.02	0.012	rs201274224	5.29	0.00	.	MISSENSE(S544A_PIK3CD)
PIK3CD	N235	0/1	chr1	9780836	T	G	0.02	0.012	rs201274224	5.29	0.00	.	MISSENSE(S544A_PIK3CD)
ICOSLG	N232	0/1	chr21	45649510	A	G	0.01	NA	rs13048869	-0.871	.	.	MISSENSE(L442P_ICOSLG)
ICOSLG	N233	0/1	chr21	45649510	A	G	0.01	NA	rs13048869	-0.871	.	.	MISSENSE(L442P_ICOSLG)
ICOSLG	N234	0/1	chr21	45649510	A	G	0.01	NA	rs13048869	-0.871	.	.	MISSENSE(L442P_ICOSLG)
ICOSLG	N231	0/1	chr21	45649785	C	A	0.07	NA	.	1.28	.	.	MISSENSE(W350C_ICOSLG)

TNFRSF13B	L287	0/1	chr17	16852187	A	G	1	0.999	rs34557412	4.96	0.01	0.00	MISSENSE(C58R_TNFRSF13B)
TNFRSF13B	N231	0/1	chr17	16852187	A	G	1	0.999	rs34557412	4.96	0.01	0.00	MISSENSE(C58R_TNFRSF13B)
TNFRSF13B	L297	1/1	chr17	16852187	A	G	1	0.999	rs34557412	4.96	0.01	0.00	MISSENSE(C58R_TNFRSF13B)
PIK3R1	N205	0/1	chr5	67569254	C	T	0.49	0.027	.	3.24	.	.	MISSENSE(P26L_PIK3R1)
PIK3R1	N206	0/1	chr5	67569254	C	T	0.49	0.027	.	3.24	.	.	MISSENSE(P26L_PIK3R1)
CD84	N223	0/1	chr1	160520771	T	C	0.54	0.004	rs143879677	3.08	0.00	0.00	MISSENSE(Q289R_CD84)
CD84	N224	0/1	chr1	160520771	T	C	0.54	0.004	rs143879677	3.08	0.00	0.00	MISSENSE(Q289R_CD84)
CD84	N232	0/1	chr1	160523750	G	A	0.03	0.999	rs146076557	5.25	0.00	.	MISSENSE(T78M_CD84)
PRKCD	N205	0/1	chr3	53215280	G	A	0.9	0.083	.	-4.35	0.00	.	MISSENSE(V125M_PRKCD)
PRKCD	N206	0/1	chr3	53215280	G	A	0.9	0.083	.	-4.35	0.00	.	MISSENSE(V125M_PRKCD)
PRKCD	L288	0/1	chr3	53218928	G	T	0.96	0.733	.	5.91	.	.	MISSENSE(V276L_PRKCD)

Figure S1. Sanger sequencing of the CTLA4 frameshift mutation in the N211 patient family.

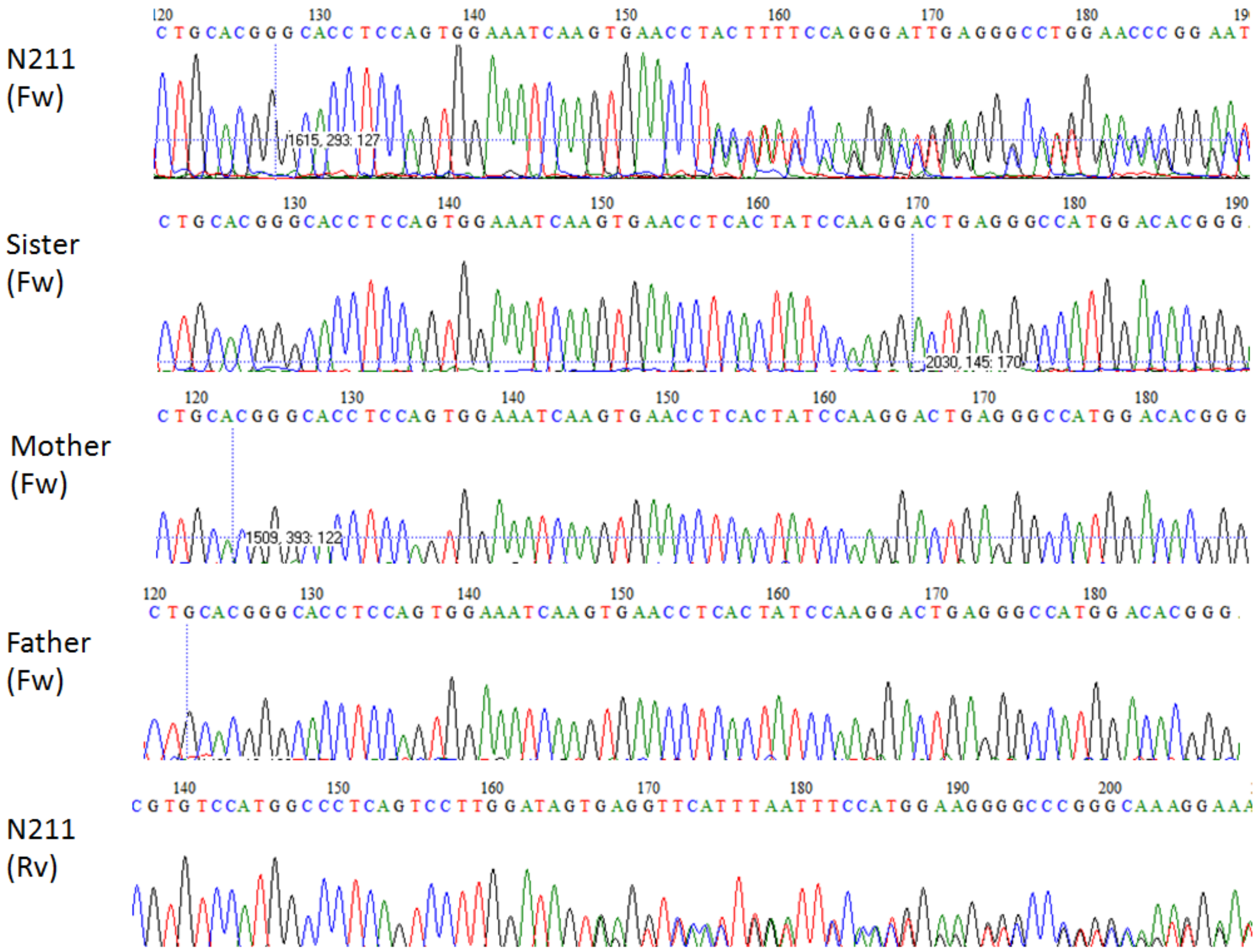

Supplement: Supplementary file 1 [file data_sheet_1.PDF]
